# Impact of C-Terminal Amide *N*-Derivatization on the Conformational Dynamics and Antimitotic Activity of Cemadotin Analogues

**DOI:** 10.3390/molecules31050825

**Published:** 2026-02-28

**Authors:** Dayana Alonso, Daniel Platero-Rochart, Pauline Stark, Leonardo G. Ceballos, Robert Rennert, Daniel G. Rivera, Julieta Coro-Bermello, Ludger A. Wessjohann

**Affiliations:** 1 Department of Bioorganic Chemistry, Leibniz Institute of Plant Biochemistry, Weinberg 3, 06120 Halle (Saale), Germany; dayananorma.alonsopalacio@ipb-halle.de (D.A.); pauline.stark@ipb-halle.de (P.S.); leonardo.gonzalezceballos@ipb-halle.de (L.G.C.); robert.rennert@ipb-halle.de (R.R.); 2 Laboratory of Synthetic and Biomolecular Chemistry, Faculty of Chemistry, University of Havana, Havana 10400, Cuba; 3 Laboratory of Computer-Aided Molecular Design, Division of Medicinal Chemistry, Otto-Loewi Research Center, Medical University of Graz, Neue Stiftingtalstraße 6/III, A-8010 Graz, Austria; daniel.platero-rochart@medunigraz.at; 4 Program Center MetaCom, Leibniz Institute of Plant Biochemistry, Weinberg 3, 06120 Halle (Saale), Germany

**Keywords:** antimitotic compounds, cemadotin, STD-NMR, conformational analysis, anticancer peptides

## Abstract

Tubulin is a heterodimeric protein composed of α- and β-subunits, which polymerize to form the cell’s microtubules. The latter are key components in mitotic spindle formation and essential targets in anticancer therapy. Compounds such as paclitaxel, tubulysins, dolastatins and synthetic analogues of these latter compounds, including cemadotin, exert their cytotoxic effects by disrupting microtubule dynamics. Previously, we reported the production and anticancer activity of a library of cemadotin analogues featuring a C-terminal tertiary amide functionalized with a variety of *N*-substituents, thus resulting in compounds occurring as a mixture of amide rotamers. Here we describe a comprehensive NMR and conformational study that provides new insights into the effect of the conformational equilibrium on the binding mode of the novel cemadotin analogues to the tubulin target. The conformational behavior of the isomer equilibrium of cemadotin’s terminal amide bond was investigated by TOCSY and ROESY NMR experiments, which allowed the identification and quantification of individual rotamer populations. A slow interconversion between the *s*-*cis* and *s*-*trans* amide rotamers was observed under standard NMR conditions (25 °C), indicating a significant energy barrier and conformational rigidity. Molecular docking and saturation transfer difference (STD) NMR experiments were performed with a representative analogue and tubulin to assess the binding mode. The results revealed that the *s-trans* rotamer is the predominant conformer in solution and exhibits a more favorable interaction with tubulin compared to the *s-cis* isomer, thus helping to understand the conformational requirements for an improved tubulin binding and the inhibition of the polymerization process.

## 1. Introduction

Tubulin is a heterodimeric protein composed of two subunits, α- and β-tubulin, which polymerize to form the microtubules in a highly dynamic process that plays an essential role in cell division by forming the mitotic spindle [1]. These structures are involved in a dynamic and continual assembly and disassembly process within the cell [1,2]. During microtubule polymerization, GTP is hydrolyzed to GDP in the β subunit, and then another GTP molecule can substitute GDP for GTP to regenerate the GTP–tubulin complex. The dynamic instability of microtubules is associated with this exchange process and can be disrupted by destabilizing agents [1,2]. Microtubules have an important role in fundamental physiological processes such as cell division, cell signaling, and intracellular trafficking in all eukaryotic cells [3]. Several compounds, including paclitaxel, tubulysins, dolastatins, and synthetic analogues of them, are known to disrupt the microtubule dynamics [4]. By doing so, such compounds affect the mitotic spindle, arresting cell division during mitosis and therefore inhibiting eukaryotic cell proliferation [5]. This process promotes apoptosis, having remarkable application in the field of anticancer therapy [6,7].

**Figure 1:**
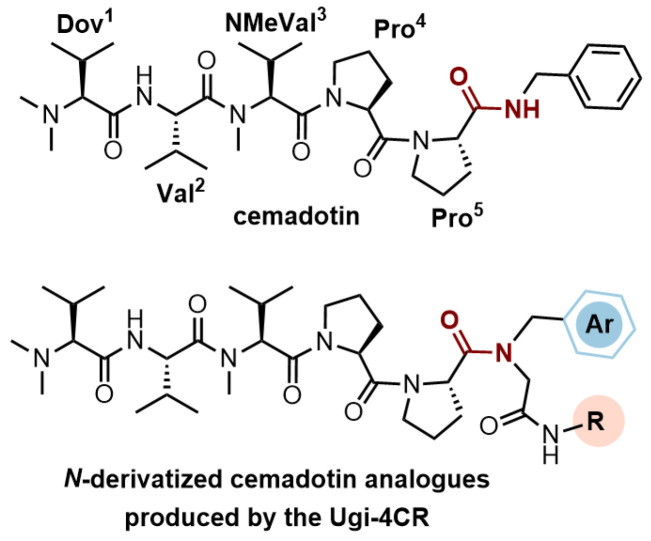
Cemadotin and backbone *N*-derivatized analogues produced by the Ugi-4CR.

We have recently described the synthesis and evaluation of the anticancer activity of cemadotin analogues using a multicomponent reaction for the diversification of the peptide backbone (Figure 1) [8]. Cemadotin (Dov^1^-Val^2^-NMeVal^3^-Pro^4^-Pro^5^-NH-Bn, Figure 1) is a highly active analogue of the naturally occurring compound dolastatin 15, but unfortunately both cemadotin and dolastatin 15 suffer from metabolic inactivation due to the proteolytic cleavage of the C-terminal fragment. To seek for more proteolytically stable analogues, cemadotin derivatives featuring a peptoid-like moiety at the C-terminal fragment were produced using the Ugi four-component reaction [9,10,11,12]. For this purpose, peptide Dov^1^-Val^2^-NMeVal^3^-Pro^4^-Pro^5^-OH was reacted with a variety of isocyanides and primary amines in the presence of paraformaldehyde to produce a library of analogues bearing a *N*-substituted cemadotin-like skeleton (Figure 1).

The biological evaluation of the cemadotin-like library proved that the incorporation of the amide *N*-substituents provoke a significant decrease (ca. 100–1000 fold) in the antiproliferative activity compared to cemadotin [8] which has a IC_50_ of about 1 nM in several cancer cell lines. Intrigued by the structural reasons behind such a loss of antiproliferative activity, we sought to conduct a spectroscopic and conformational study aiming to understand the effect of the conformational equilibrium on the binding mode of *N*-substituted cemadotin analogues to its biological target. The STD-NMR experiment was initially designed to identify small ligands from a mixture of molecules that bind to a receptor protein [13]. This method has been widely used to study tubulin interactions with several antimitotic compounds [14]. The studies have included peloruside [15], longipinane [16], HTI-286 [17], and triazolopyrimidines [18]. However, despite the large number of reported dolastatin analogues, none have been studied using this technique. This method offers many advantages, such as identifying which specific ligand conformation binds to the protein, which is critical for designing optimized derivatives.

Among the various methods based on NMR spectroscopy to determine the rotamer population, we chose to determine the ratio directly from the proton integrals in the ^1^H NMR spectrum, which has traditionally been the standard method when rotamers are observed in compounds containing tertiary amides under standard conditions (T = 298 K) [19,20,21,22]. Due to slow interchange, non-overlapping signals among conformers are identified, enabling the determination of the rotamer population without applying sophisticated methods that involve several approximations. Here, we describe a comprehensive analysis of the rotamer population in the family of Ugi-4CR-derived cemadotin analogues, along with the dynamics of the *cis-trans* equilibrium in a selected compound using metadynamics. This method aids in examining conformational changes in peptides, allowing for the investigation of different areas of the energy landscape. In addition, docking simulations of the *cis* and *trans* isomers were carried out to predict their interaction with the tubulin target. Finally, the interacting conformations were validated by a saturation transfer difference (STD) NMR experiment [23], which allowed the characterization of the rotamer mixture in complex with the αβ-tubulin receptor.

## 2. Results and Discussion

**Figure 2:**
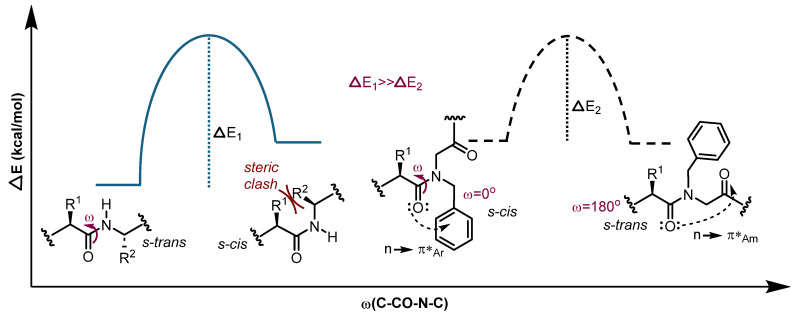
Conformational isomerism in peptides (**left**) and peptide–peptoid hybrids (**right**) considering stabilizing (e.g., n → π*Ar or the n → π*Am) and destabilizing interactions (e.g., steric) on the *s-cis* and *s-trans* conformations. Ar: aromatic and Am: amide.

The cemadotin analogues that are subject of this study feature a peptide–peptoid hybrid structure, with two consecutive prolines (Pro) preceding the *N*-substituted glycine residue resulting from the Ugi-4CR. Peptoids and peptide–peptoid hybrids are compounds characterized by dynamic effects in their NMR spectra due to the presence of one or more tertiary amides with a rather bulky *N*-substituent [22,24]. In canonical peptides, the high energy barrier (~14–20 kcal/mol) for the C-N bond rotation typically leads to non-interconvertible *s-cis* and *s-trans* amide bond rotamers [25]. However, in the case of peptoids, the additional *N*-substitution on the amide reduces the energy barrier [21,22], resulting in an amide bond isomerism characterized by a few rotational axes and a lower energy difference. These characteristics allow for the interconversion of the two configurational isomers and the frequent occurrence of both rotamers in solution (Figure 2) [21,26].

We have previously studied the conformational behavior of theUgi-4CR-derivatized bis-amide skeleton bearing a tertiary amide [27,28,29]. The relative population of the *s-cis/s-trans* rotamers depends of course on the size of the amide *N*-substituent, but besides of steric factors, other interactions such as hydrogen bonding, van der Waals or electron density donations (i.e n → π*_Ar_ or the n → π*_Am_) also define the equilibrium dynamics [30,31]. These interactions may create significant energy differences between the amide bond isomers, stabilizing a specific rotamer and restricting interconversion, which certainly influences the physicochemical and biological properties of the compound [21]. As a result, we hypothesized that the reduced activity of our cemadotin derivatives [8] could be related not only to the additional functionalization but also to the disfavored interaction of a preferred rotamer with the receptor. Our goal is to understand the relationship between the rotamer population and *cis-trans* isomerization dynamics with the antimitotic activity using STD NMR and molecular docking.

### 2.1. Analysis of the Rotamer Population by NMR Spectroscopy

The NMR spectra of the 10 Ugi-4CR-derived cemadotin derivatives analyzed in this study show a high complexity associated with the existing conformational mixture (see the SI). The signals from the peptide backbone were assigned using 2D NMR (Tables S1–S10), with the focus on the identification of the spin systems from the *s-cis* and *s-trans* rotamers. In the spectra, the slow exchange rate allowed for the individual signals from the two rotamers to be distinctive under standard NMR conditions (T = 298 K). In cases where interchangeable signals from different rotamers did not overlap with any other one, the same multiplicity was observed. A 2D ROESY experiment was performed to analyze the dipolar interactions in each molecule and to assign the spin systems of each rotamer.

**Figure 3:**
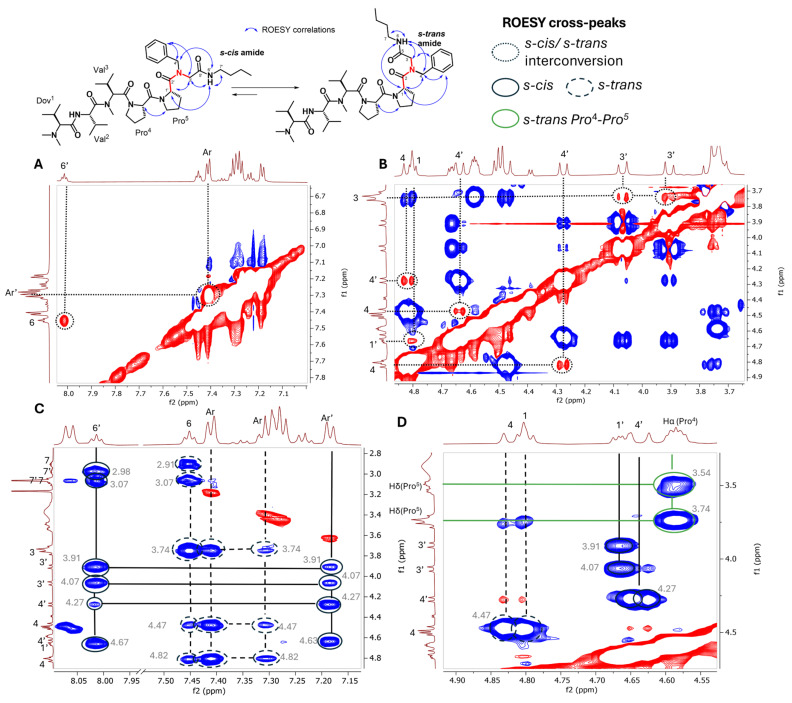
ROESY spectrum of compound **1** (DMSO-d_6_) with assigned signals corresponding to each rotamer. (**A**,**B**) Negative NOE (red)correlation peaks for interchanged signals between each rotamer. (**C**,**D**) Positive NOE (blue) correlation peaks that support the spin system of each rotamer. Dash lines and lines highlight correlations of *s-trans* and *s-cis* conformers, respectively.

The ROESY diagonal peaks have a different phase than the cross-peaks obtained by dipolar interaction within the molecule. On the other hand, peaks arising from chemical exchange (such as rotamer interchange) will have the same signal as the diagonal. This effect arises because the nuclei periodically change their Larmor frequency due to a reversible molecular dynamic process. Hence, the magnetization transfer during the mixing time is then induced by the dynamic process [19,32]. The interchanged signals of each rotamer were identified in the ROESY spectrum as correlation cross-peaks with negative NOE (red cross correlation peaks). This interconversion between several peaks can be observed in Figure 3, using compound **1** as model in this study. This is the case between the pair of signals at 8.01 ppm (6’) and 7.45 ppm (6), 7.41 (Ar) ppm and 7.28 ppm (Ar’) (Figure 3A), 4.82 ppm (4) and 4.27 ppm (4’), 4.80 ppm (1) and 4.67 ppm (1’), 4.63 ppm (4’) and 4.47 ppm (4), and 3.74 ppm (3) with 4.07 ppm and 3.91 ppm (3’) (Figure 3B), respectively. In the same spectrum, positive NOEs (blue cross-peaks) were detected and allowed the determination of the spin system of each rotamer (Figure 3C). The amide proton at 8.01 ppm (6’) couples strongly with protons at 4.67 ppm (1’), 4.07 ppm (3’), 3.91 ppm (3’), 3.07 ppm (7’), and 2.98 ppm (7’) and weakly with the proton at 4.27 ppm (4’). At the same time, the aromatic signal at 7.19 ppm (Ar’) has moderate interaction with the ones at 4.07 ppm and 3.91 ppm (3’), and an intense NOE cross-peak with the signals at 4.63 ppm and δ 4.27 ppm (4’), indicating that they belong to the same rotamer.

In contrast, the amide proton at 7.45 ppm (6) has large NOE cross-peaks with the signals at 3.74 ppm (3), 3.07 ppm and 2.91 ppm (7). This same amide proton has a weak interaction with the signals at 4.82 ppm and 4.47 ppm. In addition, the aromatic signal at 7.41 ppm (Ar) has large NOEs with those at 4.82 ppm (4), 4.47 ppm (4) and 3.74 ppm (3), whereas moderate NOEs were detected between the aromatic signal at 7.30 ppm and the ones at 4.82 ppm (4), 4.47 ppm (4) and 3.74 ppm (3) (Figure 3C).

To identify the spin system of the *s-cis* rotamer, we looked for dipolar interactions between the *H*_α_ (Pro^5^) with the methylene signals from the glycine fragment. Thus, we found a moderate NOE between the signal at 4.67 ppm (1’) with those at 4.07 ppm and δ 3.91 ppm (3’) (Figure 3D). On the other hand, for the *s-trans* isomer, *H*_α_ (Pro^5^) is closer to the methylene group from the benzyl fragment (4). In Figure 3D, a high-intensity NOE cross-peak was detected between the signal at 4.80 ppm (1) and that at δ 4.47 ppm (4), indicating a stable interaction.

The two prolines in both conformers have *C*_β_ chemical shifts between 27 and 28 ppm in DMSO-d_6_ and between 29 and 30 ppm in CD_3_OD, whereas the values of the *C*_γ_ group are around 24–26 ppm, indicating the *s*-*trans* isomer ΔC_βγ_ ≈ 4 ppm for the Pro^4^-Pro^5^ system [33]. This fact is confirmed by the close spatial proximity of *H*_α_ (Pro^4^) at 4.59 ppm and H_δ_ (Pro^5^) at 3.74 ppm and 3.54 ppm (Figure 3D). Once the signals corresponding to each rotamer were identified (SI, Tables S1–S10), the integration of N*H* amide protons arising from the isocyanide moiety was employed to calculate the rotamer population for each molecule.

Table 1 shows the rotamer population determined by NMR for each of the molecules. Within this family, the *s-trans* rotamer is consistently the more populated one. Nevertheless, the complete suppression of the *s-cis* isomer was never observed. Analysis of data in Table 1 indicates that the isocyanide moiety influences the population ratio of both rotamers. In compounds with the benzylamine moiety originally found in cemadotin, long linear hydrophobic chains (e.g., compounds **1**, **2**, **5** and **6**) increase the population of the *s-cis* rotamer up to around 45%. In contrast, compounds derived from benzylamine but bearing bulkier substituents arising from the isocyanide moiety (e.g., **3** and **4**) generally exhibit lower K*_s-trans_*_/*s-cis*_ values at 298 K, with a *s-cis* population of about 40%. A similar behavior was observed for *N*-methyl-cemadotin (**10**), an analogue bearing a methyl *N*-substituent at the C-terminus amide that was produced for comparison. For this molecule, the population of the *s-cis* rotamer was higher than expected (39%), considering the small size of the amide *N*-substituent. As previously reported by us [6], the antimitotic activity of all these molecules bearing the *N*-substituted benzyl amide was various orders of magnitude lower than that of cemadotin.

Among the compounds analyzed, derivatives **8** and **9** showed the highest population of the *s-trans* isomer (63%). However, compound **8** was the only one with activity in the nanomolar range across the three cancer cell lines tested, while the cytotoxicity of compound **9** was in the micromolar range, like compounds with the benzyl moiety (IC_50_ 2–8 μM) [8]. Since the populations of the *s*-*cis* and *s*-*trans* rotamers for these two derivatives were the same, the difference in the activity here is just related to the different nature of the aromatic ring in each structure. The different behavior could be due to the electron-rich nature of the furan ring, which differs from electron-poorer thiazole, as this could influence the possibility of forming π-π interactions within the active site [6].

### 2.2. Conformational Search and Energy Barrier

Among the benzyl derivatives, compound **4** had the highest differences between the populations of the two isomers (Table 1). As a result, this compound was chosen as model to describe the conformational differences in this library. A conformational search for compound **4** using Conformer-Rotamer Ensemble Sampling Tool (CREST) was performed. This tool is based on a semiempirical quantum chemical method, which efficiently explores low-energy molecular chemical space [34]. The analysis revealed that, starting from the *s-cis* conformation, 2.45% of the *s-trans* rotamer are found. In contrast, when starting from the *s-trans* conformation, only 0.85% of the *s-cis* conformation was detected (Figure S30). This lower interconversion from the *s*-*trans* conformer into the *s*-*cis* conformer, rather than the other way around, suggests a higher stability of the *s*-*trans* structure and agrees with the experimental data reported here.

**Figure 4:**
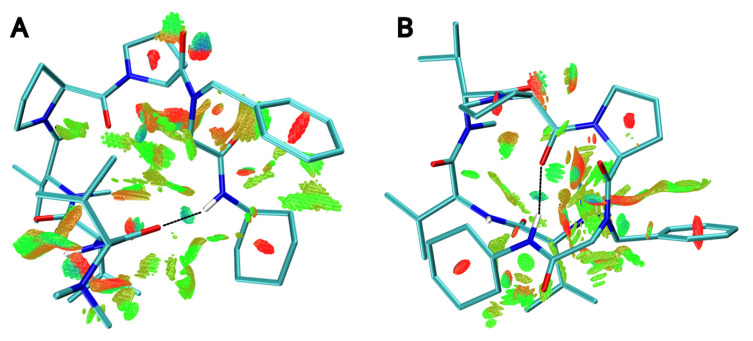
Non-covalent intramolecular interactions of compound **4** in (**A**) the *s-cis* and (**B**) the *s-trans* configuration. The black lines highlight the hydrogen bonds in each structure. Intramolecular interactions are represented in a surface with a gradient color from red to green. Repulsive interactions are displayed in red, while attractive interactions due to van der Waals contacts are represented in green. The yellowish surface corresponds to weak interactions.

To confirm this result, we decided to further optimize each isomer to study their intramolecular interactions and the conformational pathway between the structures. The optimization was carried out at DFT level, and the *s*-*trans* conformation was observed to be ca. 3.5 kcal/mol more stable than the *s-cis*. A qualitative analysis of the non-covalent interactions showed the presence of a hydrogen bond between the additional amide introduced in the Ugi-4CR and either the carbonyl group from the dolavaline residue for the *s-cis* (Figure 4A) or the carbonyl group from Pro^4^ residue for the *s-trans* (Figure 4B). The main difference between the two conformers was the larger number of attractive van der Waals interactions found in the *s-trans*, which correlates with the lower energy found for this conformation.

To better characterize the conformational interconversion, we decided to perform a well-tempered metadynamic simulation [11,35]. This method is useful to study conformational changes in peptides, such as the *cis–trans* rotamer interconversion, as it allows the system to escape the local minimum and explore different regions of the energy surface. We applied a biased potential to a collective variable, in this instance, the *N*-derivatized amide in which the *cis-trans* interconversion takes place. This allowed us to have an estimation of the free energy surface, and the energy barrier associated with the *s-cis*/*s-trans* conformational change.

**Figure 5:**
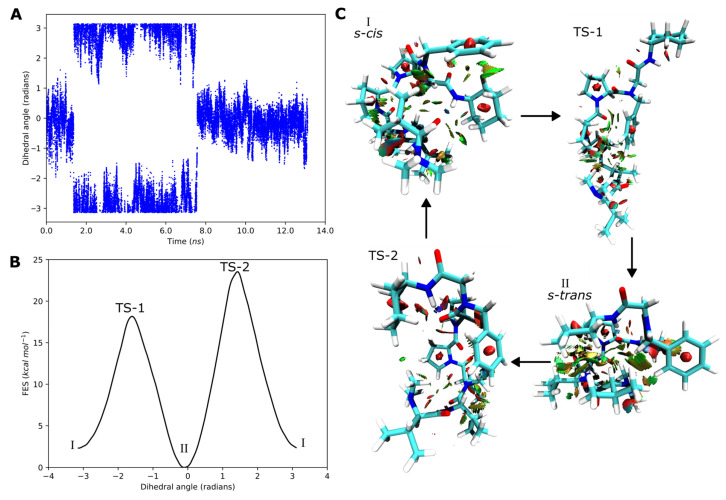
(**A**) Values of the dihedral angle explored by the simulation and (**B**) the reconstructed free energy surface. (**C**) Structure of the *s-cis*, *s-trans*, and transition conformations TS-1 and TS-2 found during the simulation. Intramolecular interactions are represented in a surface with a gradient color from red to green. Repulsive interactions are displayed in red, while attractive interactions due to van der Waals contacts are represented in green. The yellowish surface corresponds to weak interactions.

As shown in Figure 5, a simulated time of 13.2 ns was enough to explore the regions corresponding to the *s-cis* and the *s-trans* interconversion. The first transformation was observed before 2 ns of simulation and involved a change of ~3 radians (180 degrees) of the dihedral angle as expected (Figure 5A). The reconstructed free energy surface (Figure 5B) shows energy barriers of 18.2 (TS-1) and 23.5 kcal/mol (TS-2) for the *s*-*trans*/*s*-*cis* conformational shift. As can be observed in Figure 5C, the hydrogen bond observed before was disrupted in both transition structures. In addition, those conformations displayed a reduced number of attractive interactions (green surfaces) and, at the same time, a larger number of repulsive clashes (red surfaces) were observed (Figure 5C), which justifies the increase on the energy surface during the transition.

### 2.3. Docking Simulations

Microtubule polymerization is affected by several small peptides that inhibit GTP hydrolysis and/or nucleotide exchange. This process occurs by blocking the proper alignment of the catalytic residues and producing further hindrance during the polymerization process [6]. The tubulin peptide binding cavity is partially overlapping with the vinca alkaloid domain. Dolastatins, tubulysins, spongistatins, and cryptophycins are the best studied anticancer agents binding to the “peptide-site” of the vinca domain [7].

The reduced cytotoxic activity of the mixture of rotamers of our synthetic peptoids could be related to an unfavorable contribution of one of them in binding to the target. Knowing how the rotamer population affects the binding to tubulin can help us to understand the observed reduction in cytotoxicity. Two PDB structures were considered for the docking simulation, PDB: 4x1y [36] and PDB: 4zol [37], which featured co-crystallization with a dolastatin 10 analogue and tubulysin M, respectively. Although there is a high conservation of the 3D structure of the ‘peptide’ pocket near the vinca domain, this cavity is wider for the 4zol structure, which made us select this model, as it could better accommodate the compounds under study.

Redocking experiments on this system showed a similar binding mode of the docked tubulysin M and the respective co-crystalized ligand, indicating that the chosen parameters for the docking simulation were suitable. Afterward, the 10 cemadotin analogues, including their *s-cis* and *s-trans* rotamers, were analyzed using docking simulations to establish their binding conformations, intermolecular interactions, and binding energy. A summary of the number of obtained binding modes, their population among the 20 independent docking simulations, and their mean energy are shown in Table S11 (see the Supplementary Materials). As a result, the compounds screened so far showed high affinity for the peptide domain with negative binding energies (<−7 kcal/mol) and a low number of different binding modes (RMSD higher than 2.5 Å). These criteria are known as the Rosenfeld criteria for potential binders [38].

The different binding modes obtained for the *s-cis* and *s-trans* rotamers were clustered using AuPosSOM [39,40]. This program grouped the ligands considering the poses’ contact fingerprint with tubulin. The docked structures of two peptides that have the same mode of action, cemadotin and dolastatin 15, were also included in the analysis. The binding modes were clustered in 12 groups which represent different contact fingerprints (Figure S31). Binding modes in the same group have similar interaction patterns with tubulin. The summary of the fingerprint-based clustering and the interactions with the active site are listed in Table S11.

**Figure 6:**
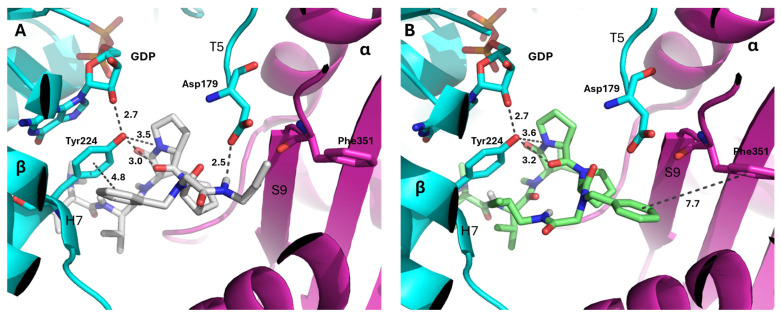
Binding modes of compound **1** in (**A**) *s-trans* (gray sticks) and (**B**) *s-cis* (green sticks) conformation. The β subunit is represented in cyan, while the α is represented in magenta. Intermolecular interactions with tubulin are displayed as black dotted lines, and distances are in Å. Key residues in the active side are highlighted in sticks. Guanosine-diphosphate (GDP) is also represented in sticks. Nitrogen: dark blue, oxygen: red.

According to the fingerprint map (Figure S32), groups 3 and 4 showed a strong interaction with β:Tyr224, the most critical residue in the active site. Especially in group 3, the interaction intensity with β:Tyr224 reached 8–11 units, the highest value among the analyzed compounds. As a general trend, the 3D structure of the docked compounds in this group showed a very conserved binding mode, independently of the configuration *s-cis* or *s-trans* (Figure S33). For example, in the case of compound **1**, the main difference between its two rotamers was the position of the aromatic ring. This fragment was oriented towards the β:Tyr224 on the *s-trans* rotamer and towards the opposite direction (towards the α:Phe351) for the *s-cis* isomer (Figure 6). These binding modes display different interactions with tubulin.

A contact-based analysis of compounds in each group was carried out using the program BINANA [39,40]. The interaction with the active site occurred mainly through hydrophobic residues or polar uncharged amino acids (Table S11). Generally, these compounds conserved the interactions with the β subunit: Gln11, Tyr210, Pro222, Tyr224, and the α subunit: Leu248; but, the backbone modification increased the interactions with many residues in the β subunit: Pro175, Lys176, Val177, Ser178, Asp179, and the α subunit: Phe351, Asn329, Ile332. Expectedly, the Pro-Pro feature in the *s-trans* configuration is necessary to maintain the turn that improves the contact with the active site.

**Figure 7:**
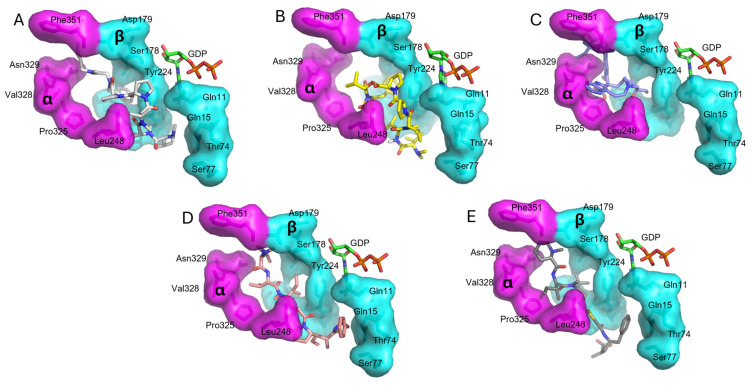
Binding mode of (**A**) compound **1**_s-trans, (**B**) dolastatin 15, (**C**) vinblastine, (**D**) soblidotin, and (**E**) tubulysin M in the peptide domain of tubulin. The surface of α-tubulin and β-tubulin is highlighted in magenta and cyan, respectively. Ligands are represented in sticks. GDP is also represented in sticks. Nitrogen: dark blue, oxygen: red.

The docked structures and docked-dolastatin 15 have different binding modes in comparison with vinblastine, soblidotin (a dolastatin 10 analogue) and tubulysin M. The *N*-terminus of the ligands, e.g., compound **1**_s-*trans* (Figure 7A), is directed to β:Gln15 and Thr74 of the peptide domain next to the vinca domain in tubulin, while the *C*-terminus occupies the gap between α:Phe351 and Asn329. The extended interaction of the synthetic compounds is not observed in the predicted dolastatin 15 binding mode (Figure 7B), which seems to be more compact. The dolastatin 15 binding mode obtained here is in good agreement with the moderate inhibitory effect of vinblastine [41] (Figure 7C, PDB code: 1z2b) [42] whose structure only partially overlaps in the vinca domain active site. In addition, the contact surface of the obtained compounds is smaller than that of soblidotin (Figure 7D, PDB code: 3e22) [6] and tubulysin M (Figure 7E, PDB code: 4zol) [37] because the Pro^4^-Pro^5^ feature induces a turn in the structure with a less extended conformation.

### 2.4. STD NMR Determination of the Rotamer’s Affinity Towards Tubulin

To validate the docking results, we performed a Saturation-Transfer Difference NMR (STD-NMR) experiment to determine the affinity of each rotamer towards the target αβ-tubulin. STD-NMR relies on the transfer of magnetization from the protein to the protons of a bound ligand. The intensity of the signal is stronger for the ligand protons that are positioned closer to the protein. We hypothesized that this approach is suitable for the validation of the most biologically active rotamers, facilitating the future design of drug candidates that preferentially adopt the active conformation.

**Figure 8:**
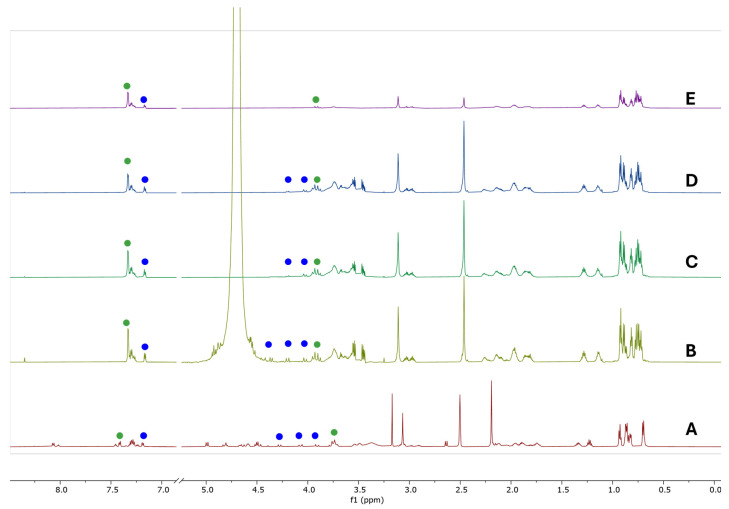
^1^H NMR spectra and STD-NMR spectra of **1**. Signal intensities fit to the highest peak in each spectrum. (**A**) Spectrum of **1** in DMSO-d_6_. (**B**) Spectrum of 350 μM of **1** in 10 mM sodium phosphate buffer pD 7.2 in 99.9% D_2_O. (**C**) Off-resonance spectrum in the presence of 7 μM αβ-tubulin under irradiation at −40 ppm. (**D**) On-resonance spectrum of the complex of **1** and tubulin obtained under irradiation at 0 ppm. (**E**) Difference spectrum showing the saturation transfer effects: in blue dots (*s-cis*) or green dots (*s-trans*).

Prior to the STD-NMR experiment, we measured the ^1^H NMR spectrum of compound **1** (Figure 8B), as well as its TOCSY and ROESY NMR spectra, in a 10 mM sodium phosphate buffer (pD 7.2 in 99.9% D_2_O) to identify the rotamers’ signals under these conditions. As can be noticed, the chemical shifts recorded in the buffer are shifted to higher frequencies (Figure 8B) in comparison to the DMSO spectrum (Figure 8A), and fewer signals are observed because all exchangeable protons disappeared in the buffer solution. Nevertheless, we were able to assign two signals from the aromatic region previously identified as *s-trans* or *s-cis*. These signals δ 7.33 (d) (Ar) and δ 7.17 (d) (Ar’) (Figure S34) are coupled with the aliphatic signals δ 3.92 (dd) (3) and δ 4.37 (d) (4’), δ 4.20 (d) (3’) and δ 4.03 (d) (3’), respectively.

According to the vendor of the tubulin (αβ-dimer), its polymerization into microtubules occurs at 5 mg/mL of tubulin in buffer with 5% glycerol and 1 mM GTP, 180 µL volume, 37 °C. Taking into account that the tubulin was prepared at a concentration of 7 μM in a 10 mM of sodium phosphate buffer pD 7.2 in 99.9% D_2_O, which was freshly prepared before measuring, and the STD experiment was conducted at 25 °C, the polymerization conditions were not met. This is essential to STD-NMR experiments because polymerized tubulin will induce broadening of the signals in the NMR spectrum, which was not observed. To establish the experimental conditions, we measured the STD-NMR spectrum of compound **1** (350 μM) without the target to ensure that no signal from the ligand is saturated during the experiment. As shown in Figure S35, the control experiment with the ligand demonstrated that no signal was detected. In addition, the ^1^H NMR of the protein (7 μM) was measured without the ligand to confirm that, at this concentration, the protein signals are not visible. However, heterodimers of tubulin are co-purified with 2 mol of guanine nucleotide per mol of αβ dimer, and the presence of this cofactor is observed at this concentration (Figure S35).

The preparation of the sample consisted in dissolving the ligand in sodium phosphate buffer pD 7.2 prepared with 99.9% D_2_O without internal standard, followed by addition of the protein to obtain a final mixture of 50:1 mol (ligand)/mol (protein). The same conditions established before were used to record the off-(Figure 8C) and on-resonance (Figure 8D) spectra for this mixture. The pseudo 2D spectrum was processed and the difference spectrum was obtained (Figure 8E). As observed, the signals corresponding to the cofactor are no longer detected in the difference spectrum.

The importance of the microtubule–ligand complex is related to the ligand mechanism towards microtubule dynamics and the location of its binding site. For example, while the taxol-binding site only exists in the formed microtubules, it has been reported for vinca-site ligands that upon binding to the tubulin dimer, the longitudinal interface is constrained to a curve, which prevents the curved-to-straight transition of tubulin necessary for its incorporation into microtubules [42]. A similar behavior is expected for vinca domain peptide ligands. We have previously reported that compound **1** inhibited microtubule polymerization [8]. Considering that the peptide-site is more extensively over the β1-subunit, there are fewer structural differences between the binding site of the polymerized or depolymerized tubulin during the STD-NMR experiment. Accordingly, the STD-NMR experiment with the depolymerized form seems to be a suitable approach for describing the interaction of compound **1** with the microtubules.

The binding of the ligand induces the dissociation of the guanosine nucleotide (multiplets at 3.77 ppm, 3.64 ppm and 3.55 ppm), which occurs exclusively in the β-tubulin subunit, increasing the dynamic instability by disrupting the dimer interface. In addition, as observed in the docking pose of compound **1** (Figure 6A), the nitrogen atom and the carbonyl group of Pro^4^ are interacting with the β:Tyr224 residue, which might compete with the interaction between this residue and the GDP molecule. Most of the peptide signals remained in the difference spectrum. However, several signals in the 5.10–4.60 ppm region disappeared due to water suppression during the experiment. Consequently, it was not possible to determine whether any Hα or the methylene group from the benzyl fragment interacted with the tubulin dimer. A semi-quantitative analysis (epitope mapping) was performed to identify parts of the ligand which are in closest contact with tubulin. Within the peptide skeleton, an increase in intensity is observed at 3.11 ppm (*N*-Me-Val^3^), now more pronounced (epitope mapping 43%) than the signal at 2.54 ppm (Me_2_N from Dov^1^), which was just 25%. Strong interactions are detected for the β- and γ-protons of the valine and proline residues (region 2.25–1.6 ppm), while the δ-protons of proline (region 3.8–3.5 ppm) showed a weaker interaction with the protein.

Furthermore, the protein maintains its interactions with the aliphatic chain arising from the isocyanide moiety, i.e., multiplets at 3.00 ppm, 1.28 ppm, and 1.15 ppm, with increased epitope mapping from 48% to 64%, respectively. This suggests that the introduced *N*-functionalization enhances the interacting surface, as predicted by the docking simulation. In the spectra in Figure 8A–E, multiple signals assigned through ROESY (Figure S34) are highlighted as blue dots (*s-cis*) or green dots (*s-trans*). In the STD difference spectrum (Figure 8E), the signal at 7.33 ppm is the most intense, providing evidence of a π-π interaction between the aromatic moiety in the *s-trans* conformation of compound **1** and the Tyr β 224 residue, as predicted. A calculation of the epitope mapping at 2 s of saturation time (Table S12) showed that this signal has the higher STD effect. Simultaneously, an aromatic signal from the *s-cis* rotamer (7.17 ppm) exhibits a weaker STD effect (79%), supporting the weaker interaction of the benzyl group with the Phe α 351 residue (Figure 6B). Another significant finding was the interaction of the methylene group of the glycine residue with the pocket. The observed STD effect for the *s-trans* rotamer (δ 3.94 and δ 3.89 (d, *J =* 16.7 Hz, 2H, 3) was just 29%. In contrast, the *s-cis* rotamer signals were not observed in the difference spectrum, supporting a higher contribution of the *s-trans* rotamer to tubulin binding. The STD–NMR and docking results consistently support a preferential binding of the *s*-*trans* conformer, suggesting that this geometry provides a more favorable spatial orientation for interacting with key residues at the binding site and, consequently, is likely the biologically active form responsible for microtubule inhibition.

## 3. Materials and Methods

### 3.1. NMR Measurements

^1^H NMR, HSQC, HMBC, TOCSY and ROESY (relaxation delay of 3 s) spectra were recorded at 298K in DMSO-*d_6_* (unless otherwise noted) with a Bruker Avance Neo 700 spectrometer (Bruker, Karlsruhe, Germany) operating at 700.35 MHz (^13^C: 176.12 MHz) using a 5 mm inverse detection cryoprobe (TCI) or an Agilent VNMRS 600 NMR spectrometer (Agilent Technologies Inc., Santa Clara, CA, USA) operating at 599.832 MHz using a 5 mm inverse detection cryoprobe. Chemical shifts (δ) are reported in ppm relative to the internal standard TMS (^1^H NMR, δ 0 ppm) and to the solvent signal DMSO-*d6* (^13^C NMR, δ 39.5 ppm) or CD_3_OD (^13^C NMR, δ 49.0 ppm).

### 3.2. Conformational Search and Energy Barrier Calculations

An initial optimization of both structures was performed using GFN2-xTB [43] in ORCA v6.0 [44]. The optimized structures were taken as input for the conformational search performed in CREST v2.11.2 [34]. The most stable conformer of the *s-cis* and *s-trans* structures were optimized in ORCA v6.0 using DFT with the GGA hybrid functional B3LYP [45,46], which uses a 20% Fock exchange in an attempt to reduce the SIE (Self-Interaction Error). Its use in combination with Grimme’s D3 dispersion correction [47] has been shown to yield results in good agreement with experimental data and more accurate computational methods [48]. Additionally, the B3LYP functional has been used for small peptides and medium- to large-sized molecules [49]. The selected basis set was the Ahlrichs def2-TZVP [50,51], which is considered to be more efficient in comparison with the Gaussian type [52]. From the optimization point of view, the use of a TZ basis set is considered already sufficient, especially in combination with a hybrid functional [52]. All optimizations were performed in the gas phase. The analysis of the non-covalent interactions was done using Multiwfn [53].

The optimized structure of the *s-trans* conformer was taken as the initial structure for the well-tempered metadynamics simulation. Charges were derived using RESP model (RHF/6-31G**). The atoms were described using the general force field GAFF2 from Amber. The system was embedded in a truncated octahedron of OPC water molecules [54]. The system was minimized and heated until the desired temperature of 300 K. Afterwards, the system was equilibrated gradually reducing the restraints.

For the production steps of the simulation, the system was described using the hybrid quantum mechanics/molecular mechanics approach (QM/MM). The QM region encompassed the atoms of the *N*-functionalization with a net charge of 0 and was calculated using the self-consistent charge density functional tight binding of third order (DFTB3) [55]. We defined as the collective variable the dihedral angle difference between the *s-cis* and *s-trans* conformations. Gaussian kernels with an initial height of 1.5 kcal/mol were deposited every 500 steps using a bias factor of 10. The simulation was done in amber 20 [56] together with PLUMED v 2.8.0 [57,58,59].

### 3.3. Docking

#### 3.3.1. Ligand/Protein Preparation

Tubulin 3D structures resolved by X-ray diffraction PDB codes, 4x1y (R = 3.19 Å) [36] and 4zol (R = 2.5 Å) [37], were downloaded from the Protein Data Bank (http://www.rcsb.org). The missing sequence parts from the receptors were built using a homology model with the SWISS-MODEL server [60]. The structures were optimized using the pdb2pqr.py (Version 2.1.0) online server [61] with AMBER force field [62], and the protonation states of ionizable groups at pH = 7.4 were assigned by using PROPKA [63].

The 3D structure of tubulysin M was extracted from 4zol and used as control for redocking experiments. The 2D structures of the compounds were drawn with ChemBioDraw Ultra 22.0. Their 3D coordinates were generated with Chem3D 22.0 using a MMFF94 parametrization [64]. Then a semi-empirical Hamiltonian PM6-DH2 optimization was made with MOPAC 2016, maintaining the torsion angle C-CO-N-C at 0 degrees to optimize the *s-cis* conformation.

Receptors and ligands PDB files were converted to PDBQT format using AutoDockTools 1.5.6 [65]. The partial charges were calculated using the Gasteiger model. Non-polar hydrogen atoms were merged with the heavy atoms. In the case of the ligands, rotatable bonds were set to default using the TORSDOF utility in AutoDockTools 1.5.6. All protein residues were kept rigid. A simulation box of size of 20 × 30 × 26 Å^3^ was constructed so that it could include the ligands surface. The center of the simulation box was placed at the center of the active site −3.37, 30.37, 17.76.

#### 3.3.2. Molecular Docking Simulation and Analysis of the Receptor–Ligand Complexes

Multiple rigid molecular docking simulations were performed using the AutoDock Vina 1.1.2 program (Vina) [66]. The docking parameters were set to default except the following: exhaustiveness = 32 and num_modes = 1. Then, 20 independent runs were carried out. The predicted enzyme–ligand complexes (20 docked poses per ligand) were clustered using an RMSD < 2.5 Å. The mean binding energy (kcal/mol) was determined for each cluster. The online server of AuPosSOM (Automatic analysis of Poses using SOM) [67,68] was employed to compare the contact fingerprint similarity among the compounds. This approach is complementary to the scoring function. Then a contact-based analysis of the best-scoring pose in each group was determined using the Python-implemented computer algorithm BINANA 1.3 [40]. The most promising ligands were selected based on their binding energy and the number of common interactions with the receptor.

### 3.4. STD-NMR Experiments

A solution containing 7 μM of α,β-tubulin (porcine brain tubulin, catalogue # T-240, Cytoskeleton, Inc.), 350 µM of compound **1**, and 10 mM of sodium phosphate buffer pD 7.2 in 99.9% D_2_O was freshly prepared before measuring the NMR spectra in a Bruker AVANCE NEO 700MHz NMR Spectrometer. STD-NMR experiments were acquired at 298.1 K with 1024 transients in a matrix with 32k data points and a spectra window of 11,000 Hz. The spectra were recorded using the stddiffesgp.3 pulse sequence that includes water suppression and spinlock. Protein saturation was carried out at 0 ppm during the on-resonance and at −40 ppm for the off-resonance experiments using 50 ms Gauss-shaped pulses for a total saturation time of 2.0 s.

Control spectra were recorded under identical conditions on samples containing compound **1** or tubulin. The on-resonance and off-resonance spectra were processed independently and subtracted to provide the differential spectrum.

## 4. Conclusions

In conclusion, we have carried out a detailed NMR and conformational study that provides new insights into the rotamer population, the dynamics of the *cis-trans* isomerization and its impact on the antimitotic activity of amide *N*-derivatized cemadotin analogues. The compounds studied feature a tertiary amide produced using Ugi-4CR, which introduced new functionalities at the C-terminal fragment of the cemadotin skeleton, but also led to a decrease in the antimitotic activity. The experimental and theoretical studies aimed at determining which rotamer exhibits higher affinity for tubulin. Thus, TOCSY, ROESY and HSQC NMR experiments evidenced that all compounds coexist as a mixture of rotamers, in which the *s-trans* form is always slightly but significantly predominant. Further analysis revealed that this proportion can be affected by both the moieties arising from isocyanide or the amino components of Ugi-4CR, with the former having the highest impact. Next, a DFT-based conformational study of cemadotin analogue **4** proved that the *s-trans* rotamer is indeed some 3.5 kcal/mol more stable than the *s-cis* one, owing to a larger number of attractive van der Waals interactions. In addition, a metadynamic simulation of the same compound found that the interconversion between the two rotamers has an energy barrier of 17.5 kcal/mol. Docking simulations of rotamers of a variety of compounds suggested a higher affinity of the *s-trans* rotamers towards αβ-tubulin. For example, in the case of compound **1**, only the *s-trans* rotamer kept the essential interaction with the β:Tyr224 residue. Finally, an STD NMR experiment between compound **1** and αβ-tubulin demonstrated the results of the docking simulation since several signals corresponding to the *s-trans* rotamer, including the protons of the benzyl moiety, exhibited a higher STD effect because of a stronger interaction within the active site of the protein. Our results shed light into the structure–activity relationship of dolastatin-type anticancer compounds. They help to predict which modifications are possible and beneficial in the backbone amides of cemadotin and its derivatives.

## Figures and Tables

**Table 1:** Rotamer population of the C-terminal amide *N*-derivatized cemadotin analogues.

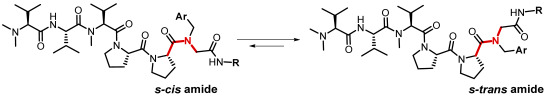						
Compound	Ar	R	ʃ_cis_	ʃ_trans_	K*_s_*_-_*_trans_*_/_*_s_*_-_*_cis_*	Population % *s-cis*/*s-trans*
**1**	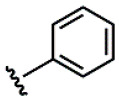	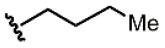	0.53	0.65	1.23	45:55
**2**	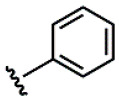	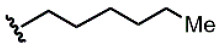	0.78	0.93	1.19	46:54
**3**	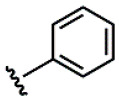	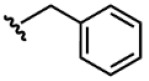	0.54	0.74	1.37	42:58
**4**	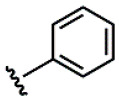	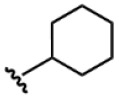	0.78	1.24	1.59	39:61
**5**	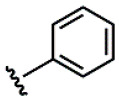	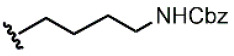	0.69	0.79	1.14	47:53
**6**	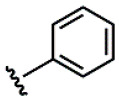	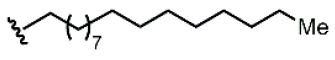	0.69	0.79	1.14	47:53
**7**	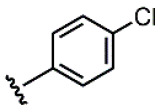	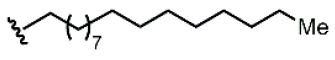	0.71	0.80	1.13	47:53
**8**	* 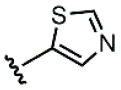 *	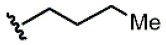	0.54	0.93	1.72	37:63
**9**	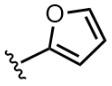	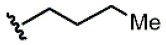	0.60	1.00	1.67	37:63
**10**	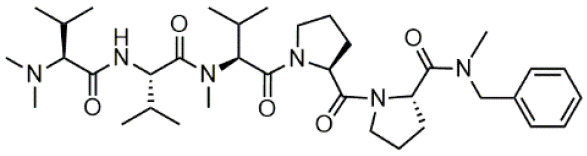		1.25	2.05	1.64	39:61

## Data Availability

The original contributions presented in this study are included in the article/supplementary material. Further inquiries can be directed to the corresponding authors.

## Supplementary Materials

The following supporting information can be downloaded at https://www.mdpi.com/article/10.3390/molecules31050825/s1, Table S1: Assignment of the ^1^H and ^13^C resonances of the *s-cis* and *s-trans* rotamers of compound **1**. Table S2: Assignment of the ^1^H and ^13^C resonances of the *s-cis* and *s-trans* rotamers of compound **2**. Table S3: Assignment of the ^1^H and ^13^C resonances of the *s-cis* and *s-trans* rotamers of compound **3**. Table S4: Assignment of the ^1^H and ^13^C resonances of the *s-cis* and *s-trans* rotamers of compound **4**. Table S5: Assignment of the ^1^H and ^13^C resonances of the *s-cis* and *s-trans* rotamers of compound **5**. Table S6: Assignment of the ^1^H and ^13^C resonances of the *s-cis* and *s-trans* rotamers of compound **6**. Table S7: Assignment of the ^1^H and ^13^C resonances of the *s-cis* and *s-trans* rotamers of compound **7**. Table S8: Assignment of the ^1^H and ^13^C resonances of the *s-cis* and *s-trans* rotamers of compound **8**. Table S9: Assignment of the ^1^H and ^13^C resonances of the *s-cis* and *s-trans* rotamers of compound **9**. Table S10: Assignment of the ^1^H and ^13^C resonances of the *s-cis* and *s-trans* rotamers of compound **10**. Table S11: Binding modes, mean energy, cluster population, fingerprint-based clustering and interactions of synthetic compounds and pattern compounds with tubulin. Table S12: Epitope mapping calculations for the mixture of conformers of compound **1**. Figure S1: Fragment of the TOCSY NMR experiment of compound **1**. The signals from the *s-cis* and *s-trans* rotamers are highlighted. Figure S2: Fragment of the ROESY NMR experiment of compound **1**. The signals that confirm the assignment of the *s-cis* and *s-trans* rotamers are highlighted. Figure S3: HSQC-NMR experiment of compound **1**. Figure S4: Fragment of the TOCSY NMR experiment of compound **2**. The signals from the *s-cis* and *s-trans* rotamers are highlighted. Figure S5: Fragment of the ROESY NMR experiment of compound **2**. The signals that confirm the assignment of the *s-cis* and *s-trans* rotamers are highlighted. Figure S6: HSQC NMR experiment of compound **2**. Figure S7: Fragment of the TOCSY NMR experiment of compound **3**. The signals from the *s-cis* and *s-trans* rotamers are highlighted. Figure S8: Fragment of the ROESY NMR experiment of compound **3**. The signals that confirm the assignments of the *s-cis* and *s-trans* rotamers are highlighted. Figure S9: HSQC NMR experiment of compound **3**. Figure S10: Fragment of the TOCSY NMR experiment of compound **4**. The signals from the *s-cis* and *s-trans* rotamers are highlighted. Figure S11: Fragment of the ROESY NMR experiment of compound **4**. The signals that confirm the assignment of the *s-cis* and *s-trans* rotamers are highlighted. Figure S12: HSQC NMR experiment of compound **4**. Figure S13: Fragment of the TOCSY NMR experiment of compound **5**. The signals from the *s-cis* and *s-trans* rotamers are highlighted. Figure S14: Fragment of the ROESY NMR experiment of compound **5**. The signals that confirm the assignment of the *s-cis* and *s-trans* rotamers are highlighted. Figure S15: HSQC NMR experiment of compound **5**. Figure S16: Fragment of the TOCSY NMR experiment of compound **6**. The signals from the *s-cis* and *s-trans* rotamers are highlighted. Figure S17: Fragment of the ROESY NMR experiment of compound **6**. The signals that confirm the assignment of the *s-cis* and *s-trans* rotamers are highlighted. Figure S18: HSQC NMR experiment of compound **6**. Figure S19: Fragment of the TOCSY NMR experiment of compound **7**. The signals from the *s-cis* and *s-trans* rotamers are highlighted. Figure S20: Fragment of the ROESY NMR experiment of compound **7**. The signals from the *s-cis* and *s-trans* rotamers are highlighted. Figure S21: HSQC NMR experiment of compound **7**. Figure S22: Fragment of the TOCSY NMR experiment of compound **8**. The signals from the *s-cis* and *s-trans* rotamers are highlighted. Figure S23: HSQC NMR experiment of compound **8**. Figure S24: Fragment of the TOCSY NMR experiment of compound **9**. The signals from the *s-cis* and *s-trans* rotamers are highlighted. Figure S25: Fragment of the ROESY NMR experiment of compound **9**. The signals that confirm the assignment of the *s-cis* and *s-trans* rotamers are highlighted. Figure S26: HSQC NMR experiment of compound **9**. Figure S27: Fragment of the TOCSY NMR experiment of compound **10**. The signals from the *s-cis* and *s-trans* rotamers are highlighted. Figure S28: Fragment of the ROESY NMR experiment of compound **10**. The signals that confirm the assignment of the *s-cis* and *s-trans* rotamers are highlighted. Figure S29: HSQC NMR experiment of compound **10**. Figure S30: Conformational search with CREST. The dihedral angle between the atoms labeled on the right was used as reference to differentiate between the *s-trans* and the *s-cis* conformations. In blue and red are the distributions of dihedral angles explored using a *s-cis* and *s-trans* respectively as a starting structure. Figure S31: Hierarchical tree map where docked compounds are clustered based on their contact fingerprint towards tubulin. Compounds docked together have a similar interaction pattern. Each cluster (0–12) is highlighted by a red number at each node. Figure S32: Contact map of the docked compounds (s-cis and s-trans conformations) which are clustered based on their contact fingerprint (Y axis) towards tubulin sequence (X axis). The color scale indicates the contact intensity average where the dark blue color corresponds to a low-intensity contact (close to zero) and in this method groups are separated by black lines. Contacts with βTyr224 is highlighted as red rimmed box. * Interaction intensity with β:Tyr224 reached 8-11 units. Figure S33: Overlay of 3D structures of the docked compounds of group 3 (Compounds **1**_*s-trans*, **1**_*s-cis*, **9**_*s-cis*, **2**_*s-cis*, **2**_*s-trans*, **8**_*s-trans*, **3**_*s-trans*), for which a conserved binding mode is observed, independently of the configuration being *s-cis* or *s-trans*. Figure S34: ROESY NMR spectra of compound **1** in 10 mM sodium phosphate buffer pD 7.2 in 99.9% D2O. *S-cis* and *s-trans* systems are observed. Figure S35: Control experiment for the ligand, compound **1** (upper spectrum) where no signal is detected as expected and tubulin (lower spectrum) where only the signals from the co-factor are detected at the tested concentration.
