# Supplementary material for: Impact of C-Terminal Amide N-Derivatization on the Conformational Dynamics and Antimitotic Activity of Cemadotin Analogues
Source: Molecules. 2026 Feb 28;31(5):825. doi: 10.3390/molecules31050825 (PMC12985878; doi:10.3390/molecules31050825)
Supplement: Supplementary file 1 [file molecules-31-00825-s001.zip › molecules-4121977-supplementary.pdf]

Supporting Information

Impact of Backbone Amide *N*-Derivatization on the Conformational Dynamics and Antimitotic Activity of Cemadotin

Dayana Alonso,[a, b] Daniel Platero-Rochart,[c] Pauline Stark,[d b] Leonardo G. Ceballos,[a] Robert Rennert,[a] Daniel G. Rivera,[a, b] Julieta Coro-Bermello,[b] Ludger A. Wessjohann.[a]

[a] Department of Bioorganic Chemistry, Leibniz Institute of Plant Biochemistry, Weinberg 3, 06120 Halle/Saale, Germany.

[b] Laboratory of Synthetic and Biomolecular Chemistry, Faculty of Chemistry, University of Havana, Havana 10400, Cuba.

[c] Laboratory of Computer-Aided Molecular Design, Division of Medicinal Chemistry, Otto-Loewi Research Center, Medical University of Graz, Neue Stiftingtalstraße 6/III, A-8010 Graz, Austria.

[d] Program Center MetaCom, Leibniz Institute of Plant Biochemistry, Weinberg 3, 06120 Halle/Saale, Germany.

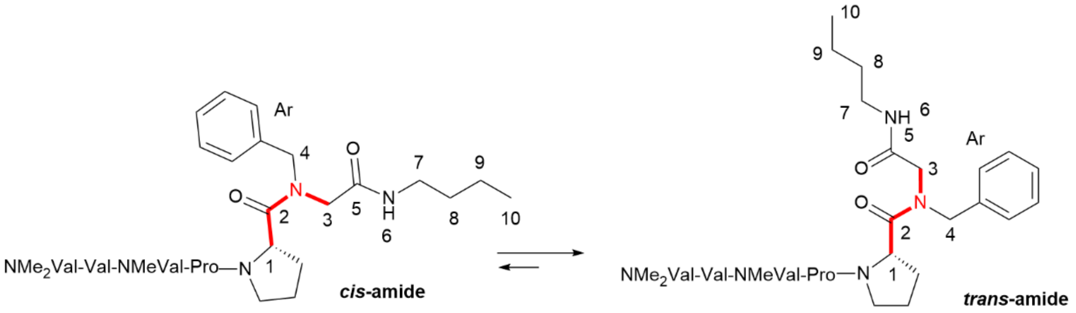

Table S1. Assignment of the ^{1}H and ^{13}C resonances of the *s-cis* and *s-trans* rotamers of compound **1**.

AA	δ_H- δ_c NH or NCH_3	δ_H- δ_c Hα-Cα	δ_H- δ_c Hβ-Cβ	δ_H- δ_c Hγ-Cγ	δ_H- δ_c Hδ-Cδ	δ_c C=O
NMe_2Val	2.19-41.2	2.64-72.7	1.88-26.8	0.87-19.5 0.70-19.2	-	170.0
Val	8.07	4.50-53.8	1.96-29.8	0.93-18.9 0.82-18.7	-	172.6
NMeVal cis	3.06-30.2	5.00-58.2	2.11-26.5	0.69-18.1 0.92-18.9	-	167.7
NMeVal trans		4.98-58.2			-	
Pro 4	-	4.59-57.5	2.17 1.76- 27.5	1.92 1.76-24.0	3.52 3.74 46.9	169.6
Pro 5 trans	-	4.80-56.5	2.03 1.73-28.3	1.86 1.96-24.5	3.74 3.49-46.9	172.1
Pro 5 cis	-	4.67-56.2	1.82 2.11-28.5	2.06 1.90-24.7	3.74 3.54 -46.9	172.3

Rotamer	δ_H- δ_c *s-cis*	δ_H- δ_c *s-trans*
1	4.67-56.2	4.80-56.5
2	172.3	172.1
3	3.91 4.07-50.0	3.74-48.7
4	4.63 4.27-49.4	4.82 4.47-51.5
5	167.6	167.2
6	8.01	7.45
7	3.07 2.98-38.2	3.07 2.91-38.2

Rotamer	δ_H- δ_c *s-cis*	δ_H- δ_c *s-trans*
8 and 9	1.34-18.1 1.34-31.0 1.22-19.5 1.22-31.2	1.34-18.1 1.34-31.0 1.22-19.5 1.22-31.2
10	0.87-13.6	0.87-13.6
Ar	7.28-127.3 7.24-126.9 7.19-127.5 137.1	7.31-128.3, 7.41-127.6 137.1

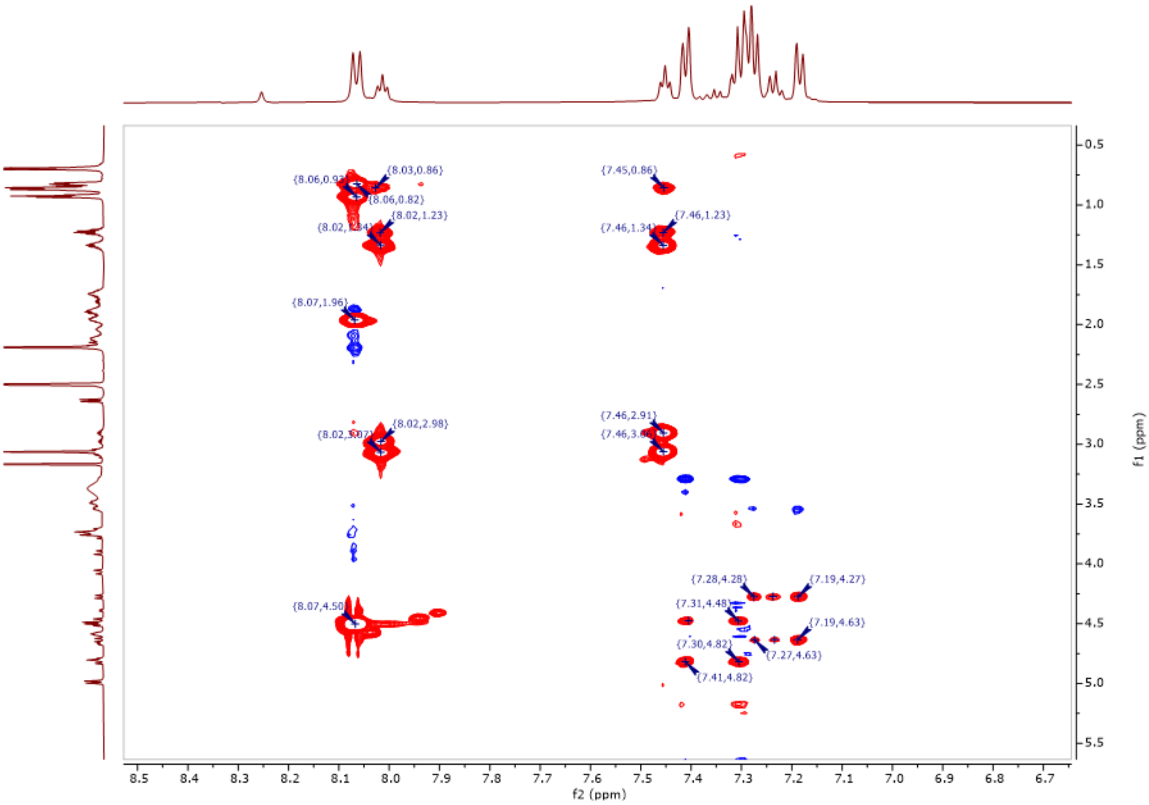

Figure S1. Fragment of the TOCSY NMR experiment of compound **1**. The signals from the *s-cis* and *s-trans* rotamers are highlighted.

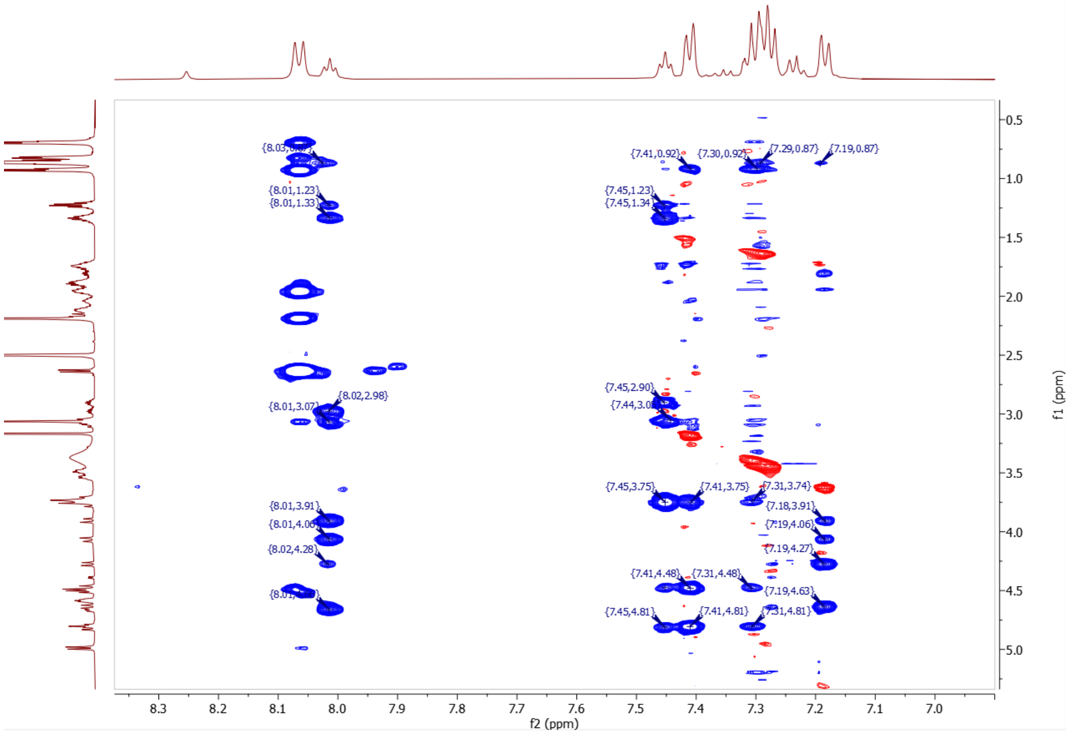

Figure S2. Fragment of the ROESY NMR experiment of compound **1**. The signals that confirm the assignment of the *s-cis* and *s-trans* rotamers are highlighted.

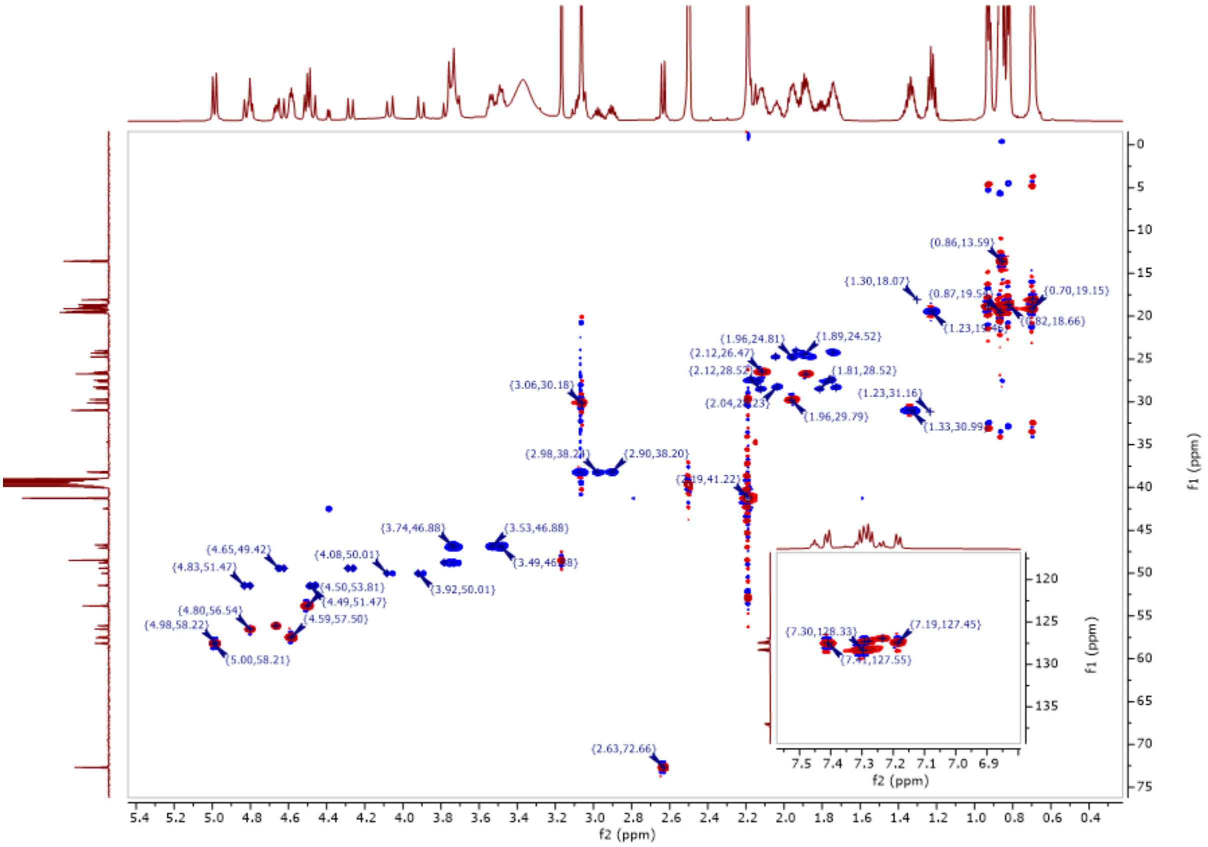

Figure S3. HSQC-NMR experiment of compound **1**.

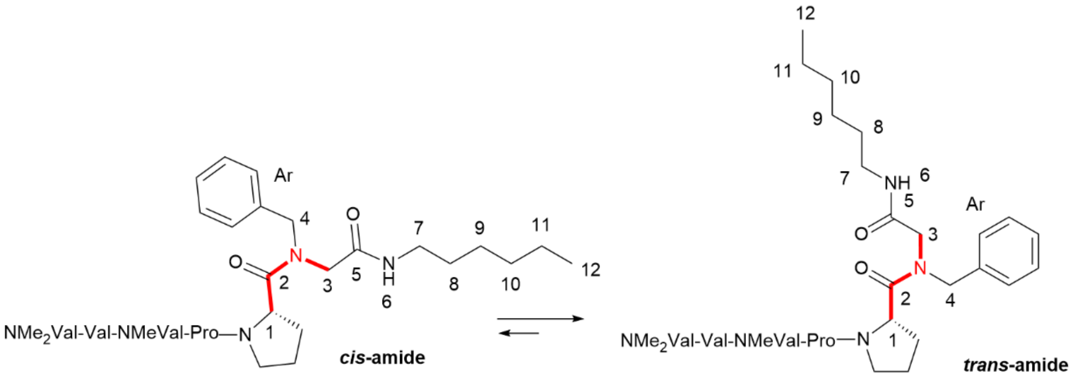

Table S2. Assignment of the ^{1}H and ^{13}C resonances of the *s-cis* and *s-trans* rotamers of compound **2**.

AA	δ_H- δ_c NH or NCH_3	δ_H- δ_c Hα-Cα	δ_H- δ_c Hβ-Cβ	δ_H- δ_c Hγ-Cγ	δ_H- δ_c Hδ-Cδ	δ_c C=O
NMe_2Val	2.20-41.3	2.65-72.5	1.89-26.7	0.87-19.4 0.71-19.0	-	170.0
Val	8.08	4.51-53.8	1.97-29.8	0.94-18.8 0.84-18.6	-	172.7
NMeVal cis	3.07-30.2	5.01-58.2	2.13-26.5	0.71-18.1 0.93-18.8	-	167.7
NMeVal trans		4.99-58.2			-	
Pro 4	-	4.60-57.5	2.18 1.76- 27.4	1.94 1.74-24.1	3.50 3.74-46.8	169.5
Pro 5 trans	-	4.82-56.5	2.05 1.73-28.2	1.87 1.97-24.7	3.75 3.49-46.8	172.1
Pro 5 cis	-	4.67-56.1	2.13 1.83 -28.4	2.06 1.91-24.5	3.54 3.75 -46.8	172.3

Rotamer	δ_H- δ_c *s-cis*	δ_H- δ_c *s-trans*
1	4.67-56.1	4.82-56.5
2	172.3	172.1
3	3.93 4.05-50.0	3.75-48.7
4	4.62-49.5	4.49-51.5
5	167.6	167.2
6	8.03	7.47
7	3.06 2.97-38.6	3.06 2.91-38.6

Rotamer	δ_H- δ_c *s-cis*	δ_H- δ_c *s-trans*
8	1.34-28.8	1.34-28.8
9	1.22-22.0, 25.9, 28.8, 30.1	1.23-22.0, 26.0, 28.8
10		
11		
12	0.87-13.8	0.92-13.8
Ar	7.19-127.4 137.2	7.31-128.2, 7.41-127.4 137.2

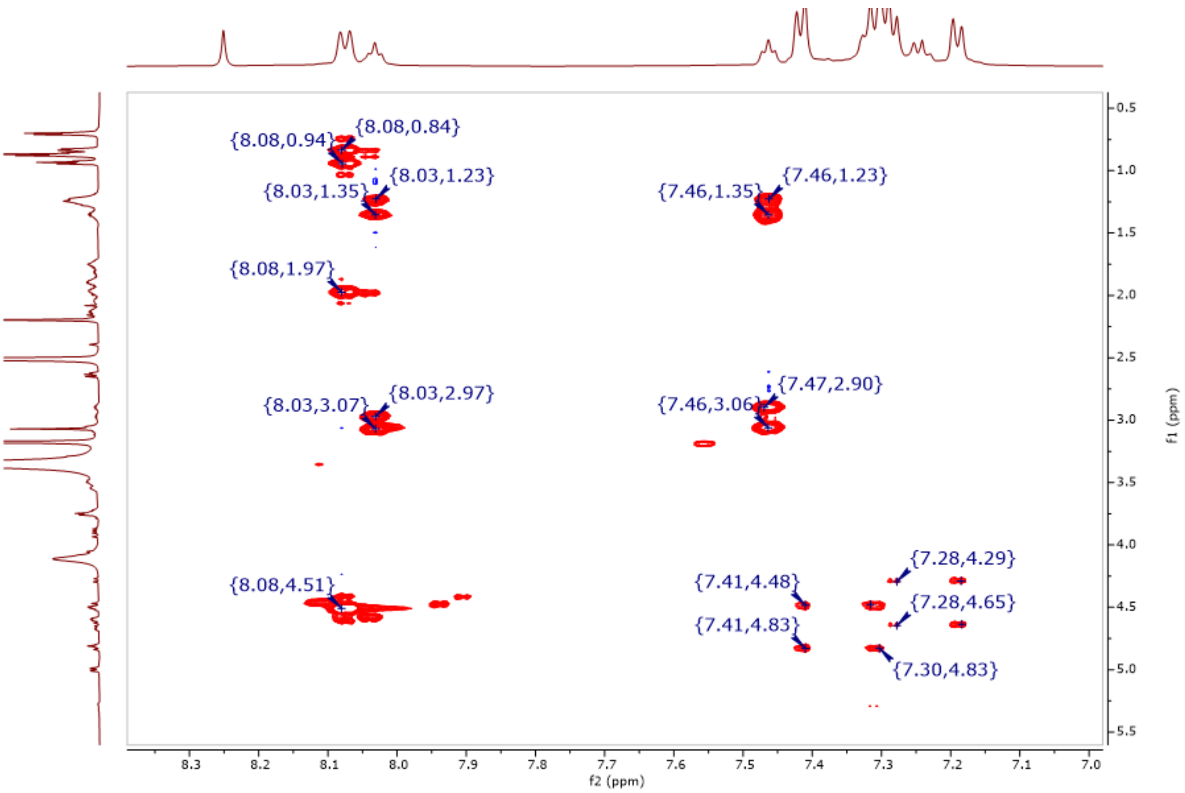

Figure S4. Fragment of the TOCSY NMR experiment of compound **2**. The signals from the *s-cis* and *s-trans* rotamers are highlighted.

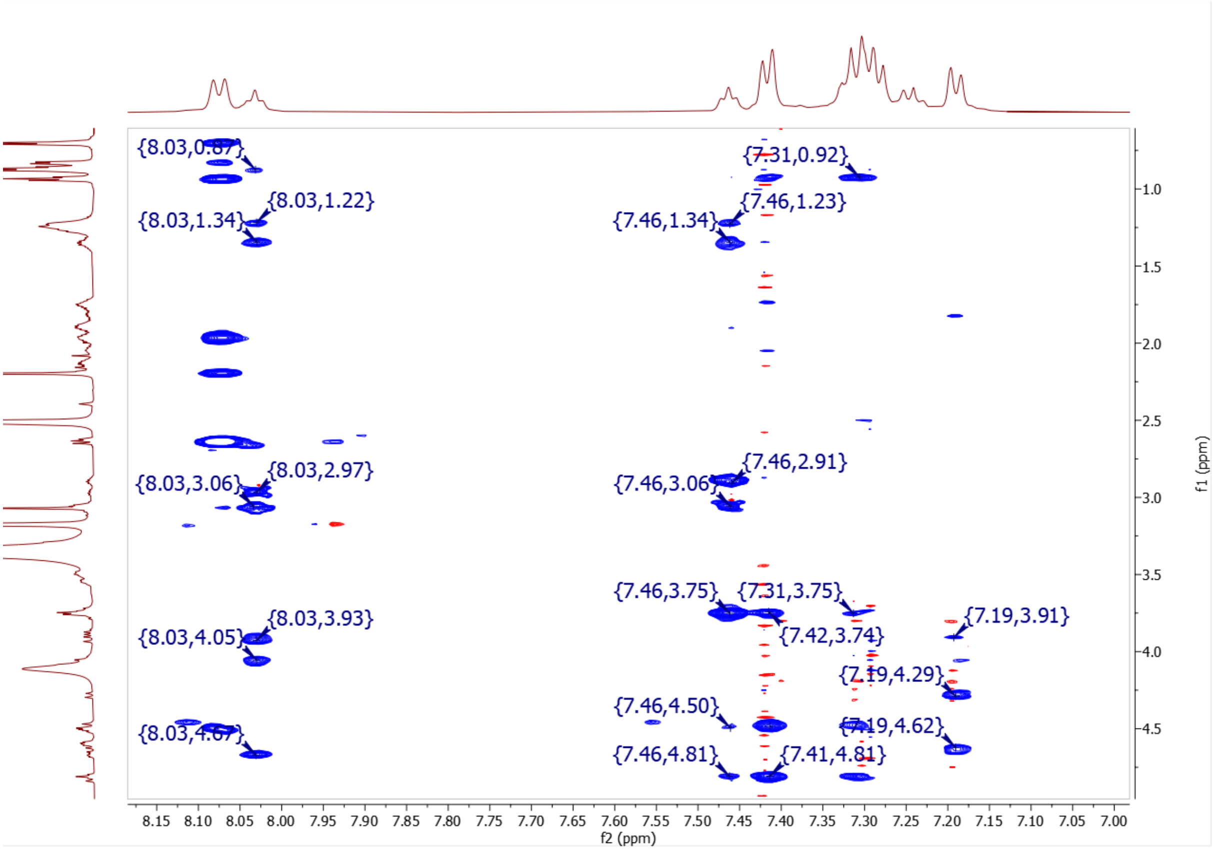

Figure S5. Fragment of the ROESY NMR experiment of compound **2**. The signals that confirm the assignment of the *s-cis* and *s-trans* rotamers are highlighted.

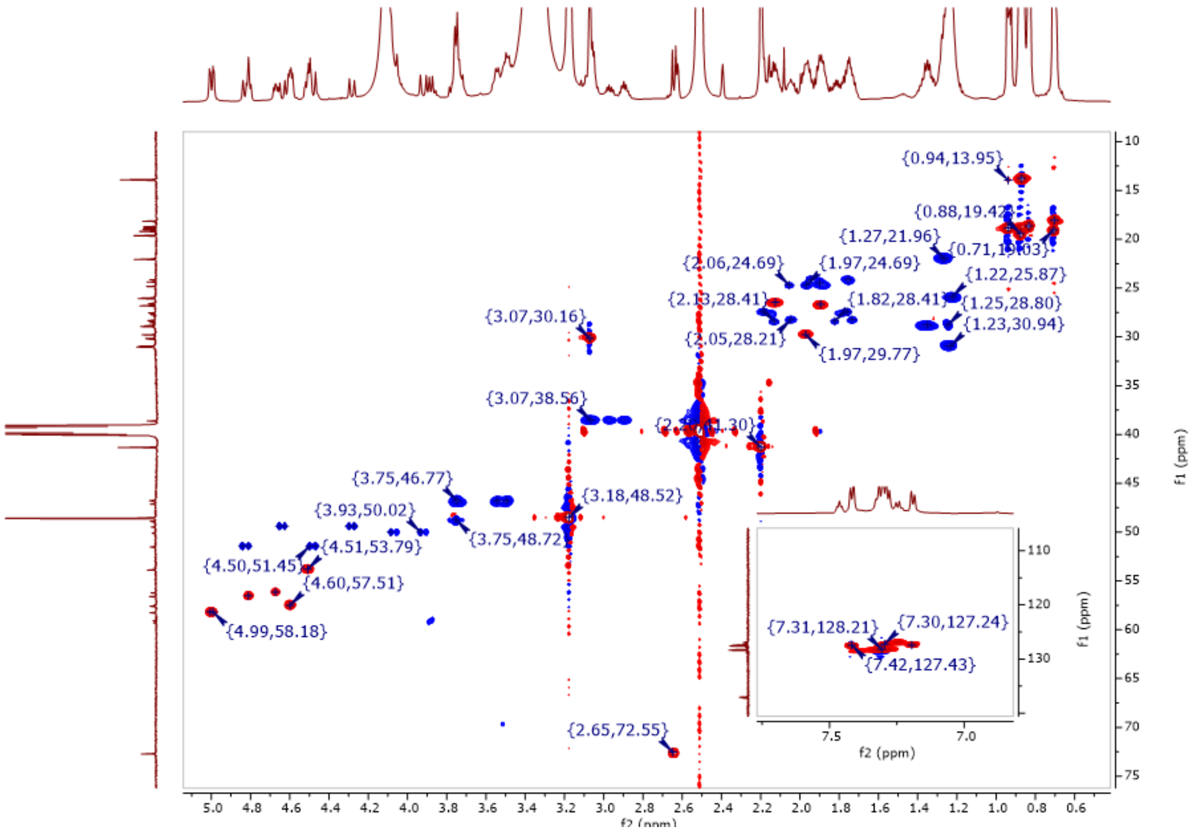

Figure S6. HSQC NMR experiment of compound **2**.

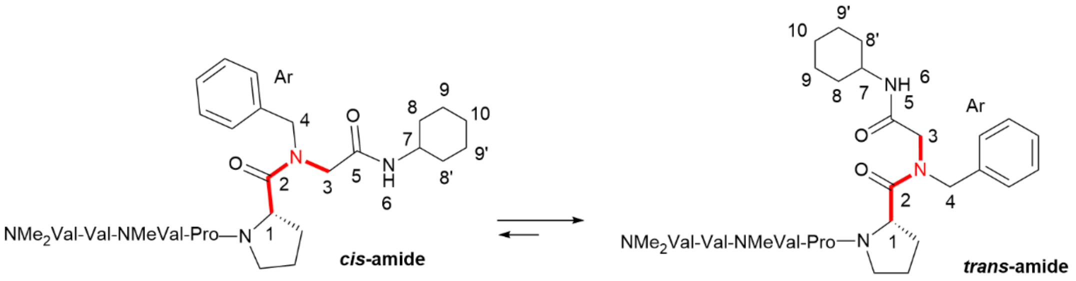

Table S3. Assignment of the ^{1}H and ^{13}C resonances of the *s-cis* and *s-trans* rotamers of compound **3**.

AA	δ_H- δ_c NH o NCH$_3$	δ_H- δ_c Hα-Cα	δ_H- δ_c Hβ-Cβ	δ_H- δ_c Hγ-Cγ	δ_H- δ_c Hδ-Cδ	δ_c C=O
NMe$_2$Val	2.20-41.5	2.64-72.8	1.89-26.8	0.88-19.6 0.71-19.2	-	169.9
Val	8.06	4.52-53.8	1.97-29.8	0.94-18.9 0.84-18.7	-	172.6
NMeVal cis	3.07-30.1	5.00-58.3	2.14-26.5	0.70-18.1 0.94-18.1	-	167.6
NMeVal trans					-	
Pro 4	-	4.59-57.5	2.21 1.77-27.5	1.94 1.77-24.4	3.50 3.74-46.9	169.5
Pro 5 trans	-	4.81-56.6	2.05 1.71-28.3	1.98 1.88- 24.7	3.75 3.49-46.8	172.1
Pro 5 cis	-	4.68-56.1	2.10 1.83- 28.5	1.91-24.7	3.55 3.75 -46.8	172.4

Rotamer	δ_H- δ_c *s-cis*	δ_H- δ_c *s-trans*
1	4.68-56.1	4.81-56.6
2	172.4	172.1
3	3.85 4.13-49.9	3.67 3.87-49.0
4	4.72 4.23-49.5	4.81 4.48-51.5
5	166.7	166.3
6	7.84	7.18
7	3.51-47.7	3.45-47.5

Rotamer	δ_H- δ_c *s-cis*	δ_H- δ_c *s-trans*
8	1.65 1.24-24.5	1.66 1.60-32.0
9	1.71-28.3	1.23-28.8
10	1.12-32.1	1.09-25.1
Ar	7.19-127.4 137.3	7.31-128.3, 7.42-127.6 137.0

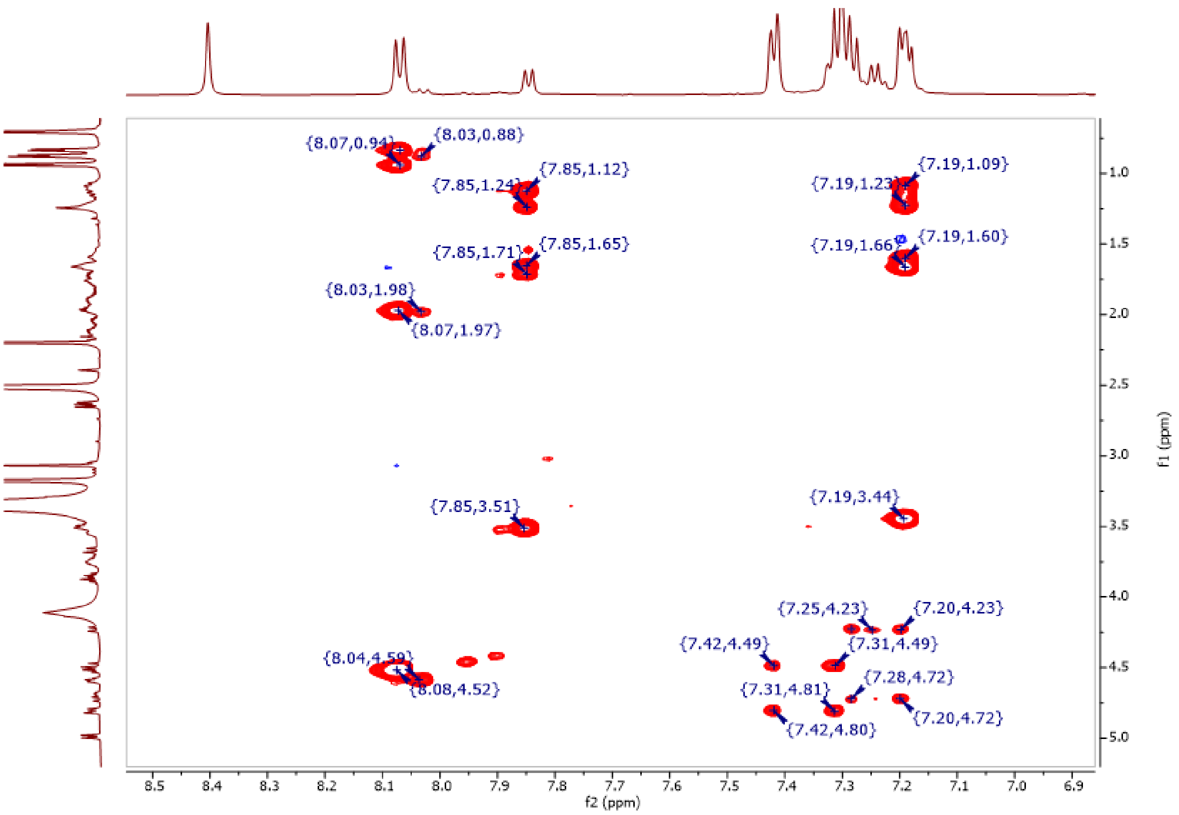

Figure S7. Fragment of the TOCSY NMR experiment of compound **3**. The signals from the *s-cis* and *s-trans* rotamers are highlighted.

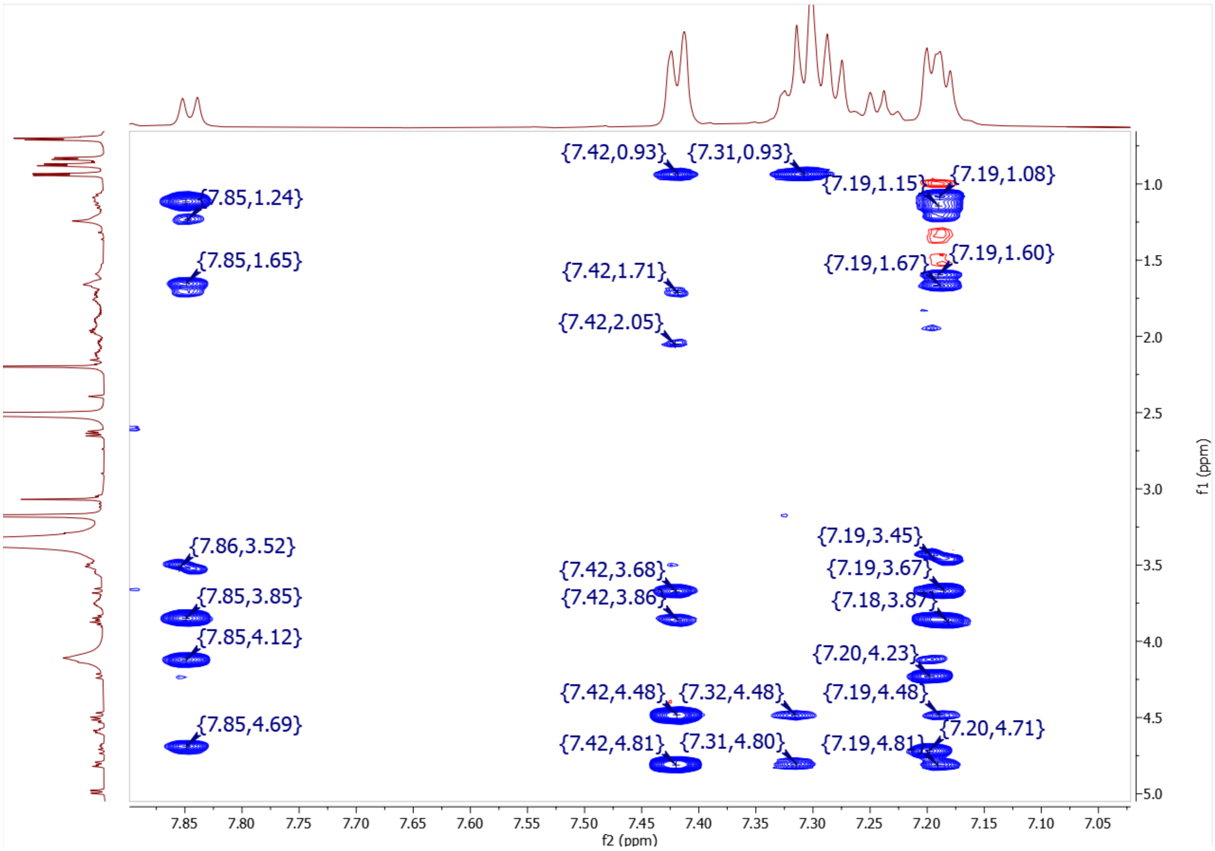

Figure S8. Fragment of the ROESY NMR experiment of compound **3**. The signals that confirm the assignments of the *s-cis* and *s-trans* rotamers are highlighted.

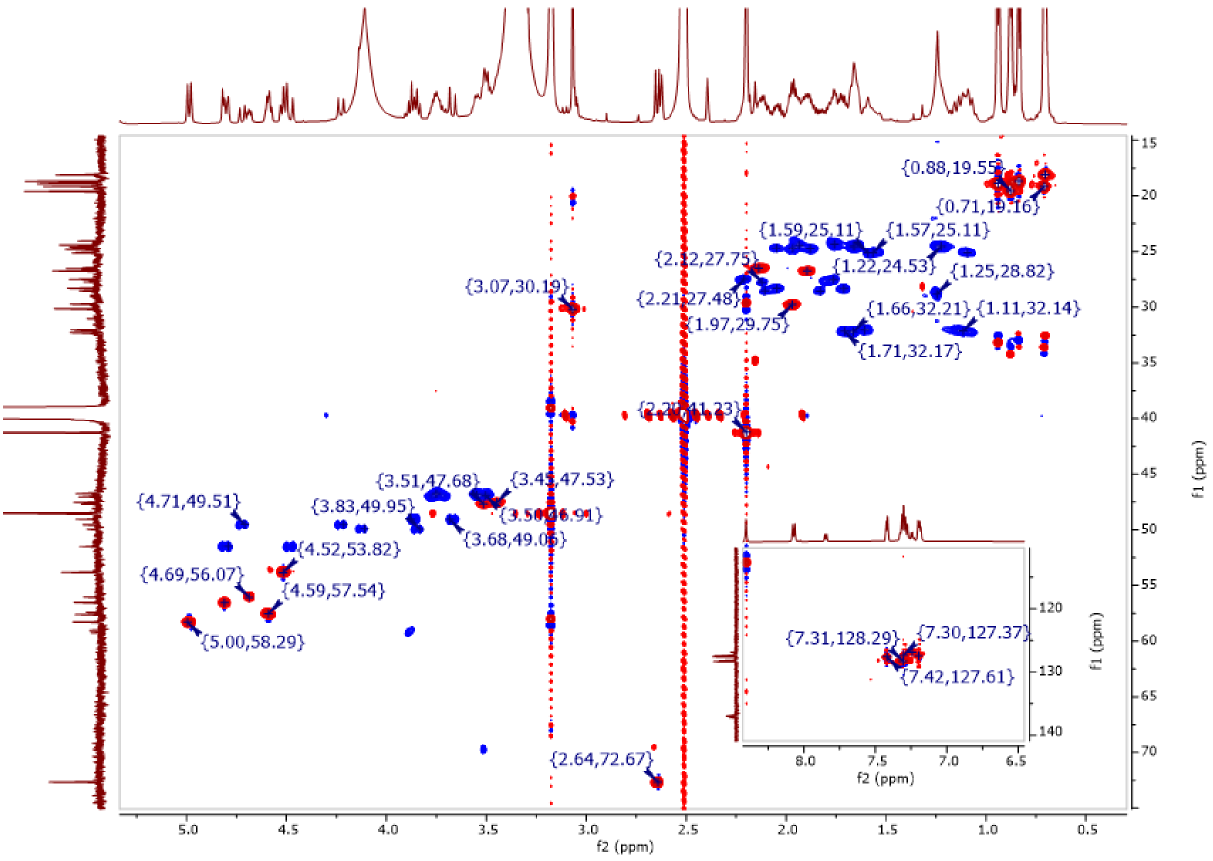

Figure S9. HSQC NMR experiment of compound **3**.

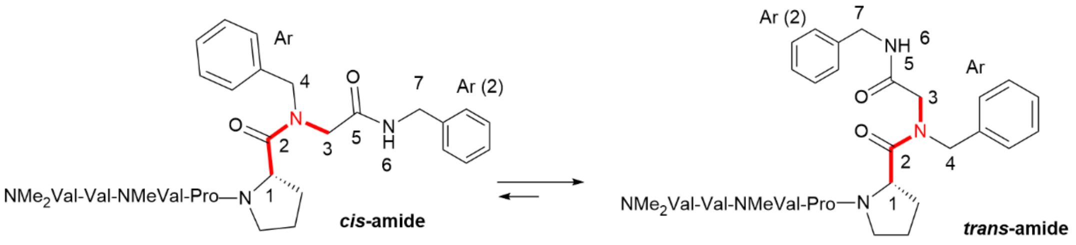

Table S4. Assignment of the ^{1}H and ^{13}C resonances of the *s-cis* and *s-trans* rotamers of compound **4**.

AA	δ_H- δ_c NH o NCH_3	δ_H- δ_c Hα-Cα	δ_H- δ_c Hβ-Cβ	δ_H- δ_c Hγ-Cγ	δ_H- δ_c Hδ-Cδ	δ_c C=O
NMe_2Val	2.20-41.2	2.63-72.7	1.89-26.7	0.86-19.5 0.69-19.1	-	170.1
Val	8.06	4.50-53.8	1.96-29.8	0.92-19.0 0.80-18.7	-	172.8
NMeVal s-cis	3.06-30.1	4.98-58.2	2.08-26.5	0.66 0.84-18.7	-	167.8
NMeVal s-trans		4.93-58.1		0.67 0.86-18.7	-	
Pro 4	-	4.60-57.5	2.15 1.69-27.4	1.63 1.76-24.3	3.50 3.74-46.7	169.5
Pro 5 s-trans	-	4.82-56.6	2.05 1.77-28.4	2.05 1.96- 24.7	3.70 3.49-46.9	172.3
Pro 5 s-cis	-	4.71-56.1	2.05 1.82-28.5	1.97 1.87-24.7	3.55 3.75 -46.9	172.6

Rotamer	δ_H- δ_c *s-cis*	δ_H- δ_c *s-trans*
1	4.71-56.1	4.82-56.6
2	172.6	172.3
3	3.93 3.96-48.7	3.75 3.73-48.7
4	4.22 4.73-49.5	4.51 4.88-51.5
5	168.1	167.9
6	8.60	8.16
7	4.20 4.33-42.2	4.23-42.0

Rotamer	δ_H- δ_c *s-cis*	δ_H- δ_c *s-trans*
Ar	7.20-126.8 137.2	7.31-128.3, 7.43-127.4 137.1
Ar(2)	7.20-127.2, 7.28-128.3, 7.30-127.4 139.1	7.20-127.4 7.24-126.9 139.2

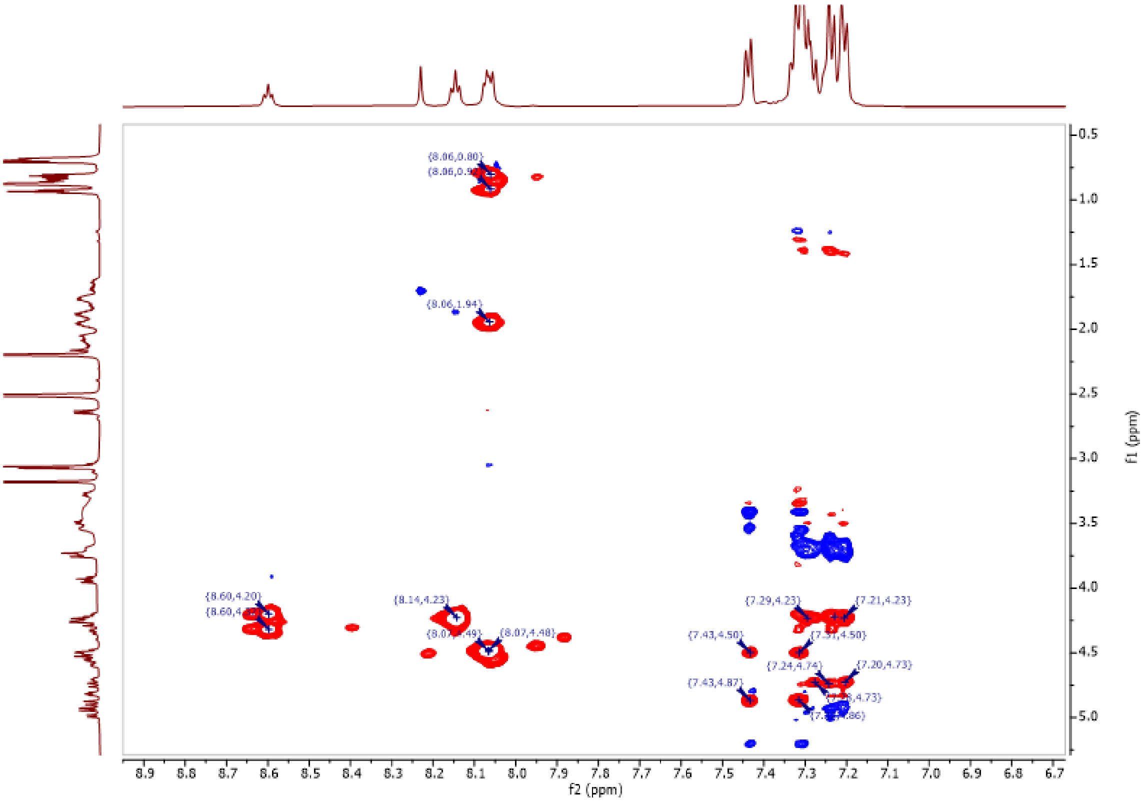

Figure S10. Fragment of the TOCSY NMR experiment of compound **4**. The signals from the *s-cis* and *s-trans* rotamers are highlighted.

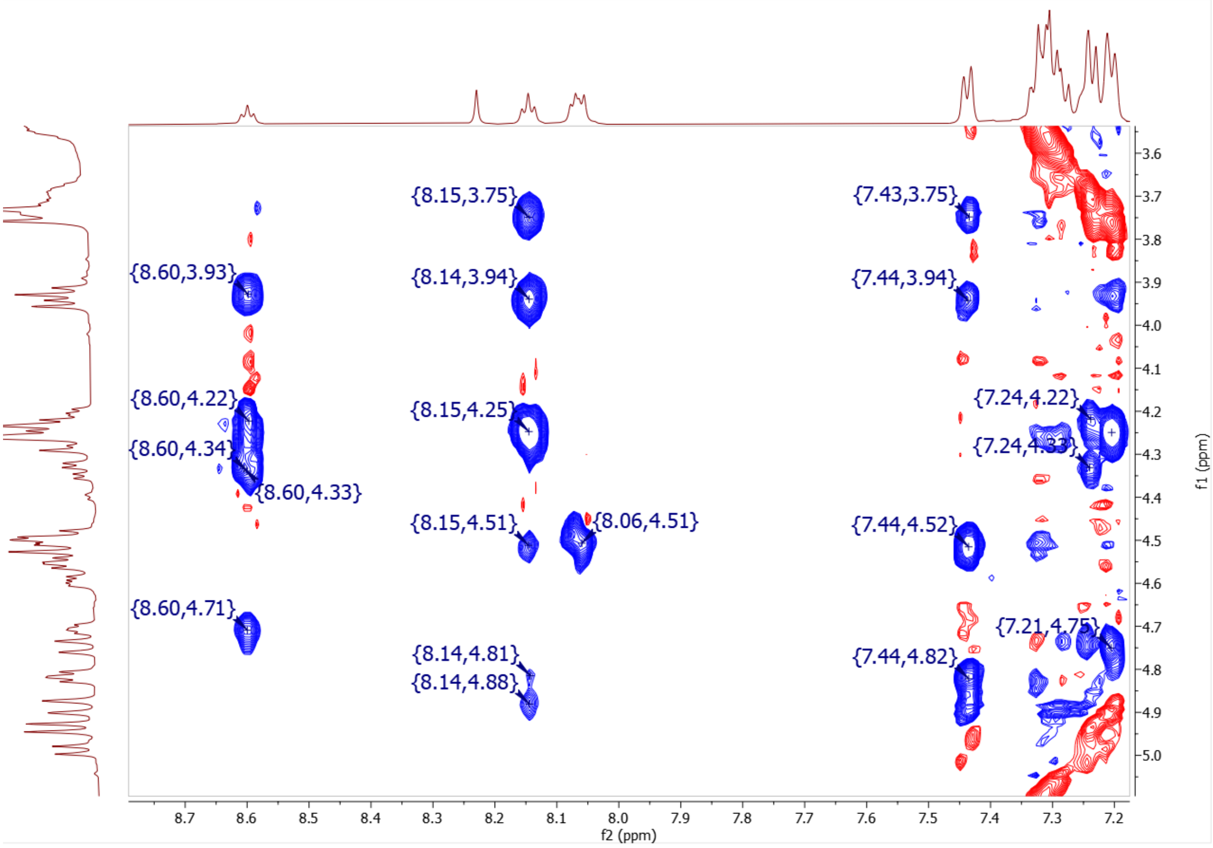

Figure S11. Fragment of the ROESY NMR experiment of compound **4.** The signals that confirm the assignment of the *s-cis* and *s-trans* rotamers are highlighted.

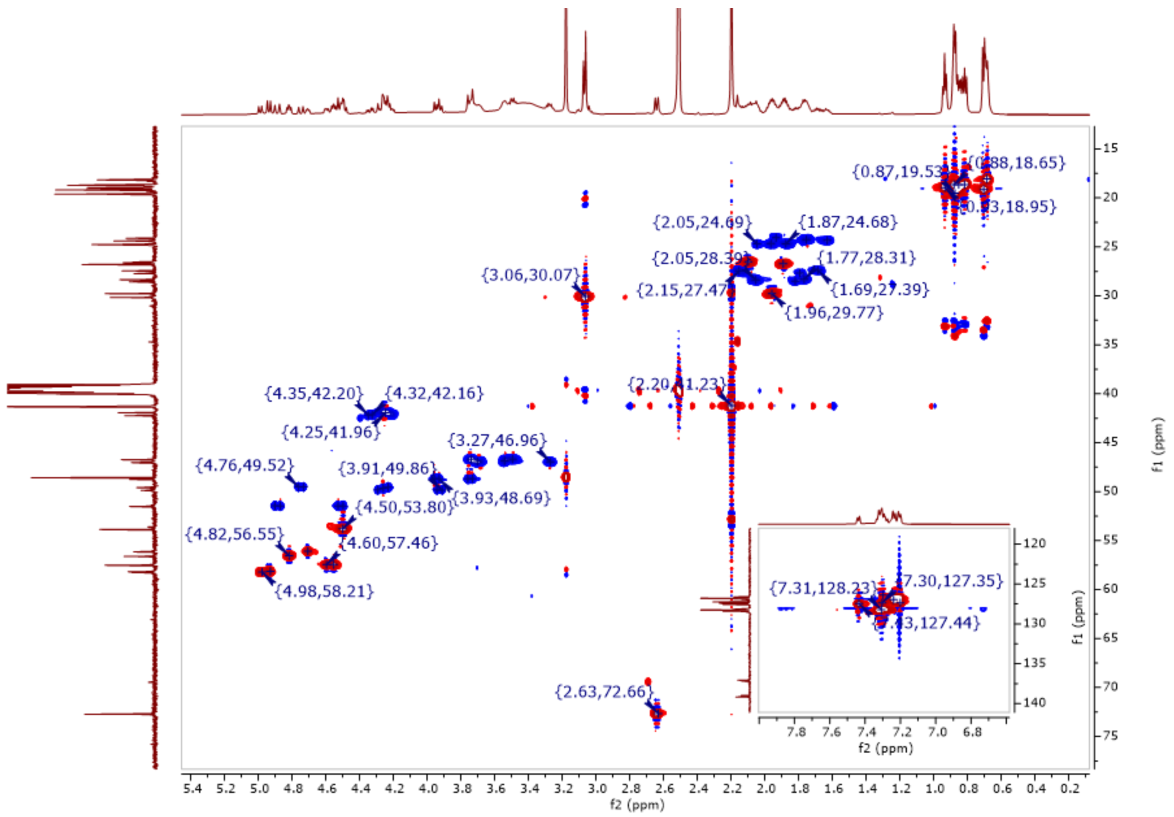

Figure S12. HSQC NMR experiment of compound **4**.

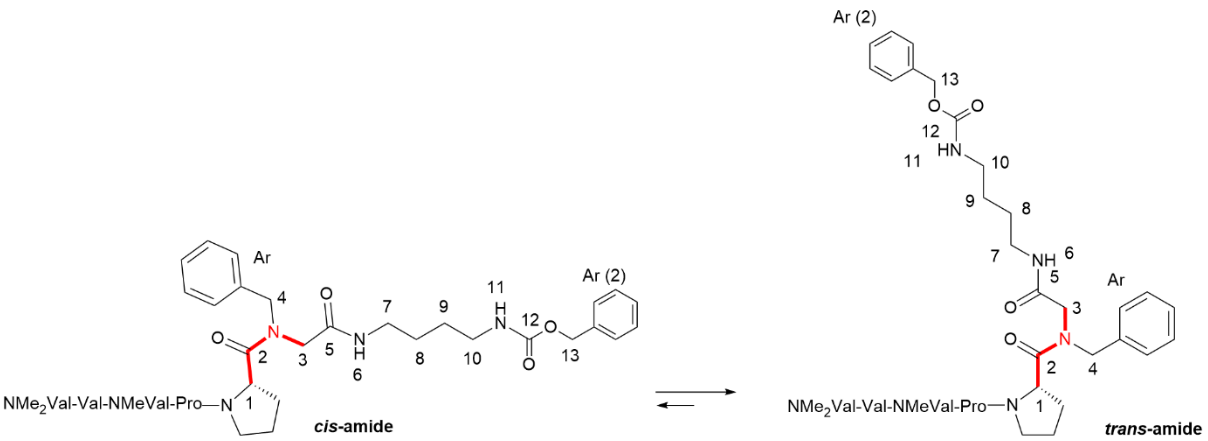

Table S5. Assignment of the ^{1}H and ^{13}C resonances of the *s-cis* and *s-trans* rotamers of compound **5**.

AA	δ_H- δ_c NH o NCH_3	δ_H- δ_c Hα-Cα	δ_H- δ_c Hβ-Cβ	δ_H- δ_c Hγ-Cγ	δ_H- δ_c Hδ-Cδ	δ_c C=O
NMe_2Val	2.20-41.3	2.64-72.7	1.89-26.7	0.87-19.0 0.70-19.2	-	170.0
Val	8.07	4.50-53.8	1.96-29.8	0.93-18.9 0.82-18.7	-	172.7
NMeVal cis	3.07-30.2	5.00-58.2	2.11-26.6	0.69-18.1 0.91-18.7	-	167.7
NMeVal trans	3.17-48.5	4.99-58.2			-	
Pro 4	-	4.57-57.5	2.13-27.6	1.92- 1.74-24.2	3.50 3.74-46.9	169.5
Pro 5 trans	-	4.80-56.5	2.05 1.74-28.3	1.86 1.95-24.6	3.75 3.49-46.9	172.1
Pro 5 cis	-	4.66-56.1	1.81 2.11-28.5	2.05 1.82-24.8	3.54 3.75 -46.9	172.3

Rotamer	δ_H- δ_c *s-cis*	δ_H- δ_c *s-trans*
1	4.67-56.1	4.81-56.5
2	172.3	172.1
3	3.90 4.07-49.9	3.82 3.64-48.5
4	4.65 4.26-49.4	4.82 4.45-51.3
5	167.6	167.3
6	8.05	7.53
7	3.08 2.97-38.2	3.05 2.93-38.2
8	1.35-26.8	1.35-26.8

Rotamer	δ_H- δ_c *s-cis*	δ_H- δ_c *s-trans*
9	1.35-26.8	1.35-26.8
10	2.97-39.9	2.97-39.9
11	6.90	7.24
12	156.1	156.1
13	5.00-65.1	5.00-65.1
Ar	7.19-127.4 127.0 137.0	7.31-128.3, 7.41-127.5 137.0
Ar (2)	7.33-127.7, 7.30-127.6, 137.2,	7.33-127.7, 7.30-127.6, 127.4, 137.2,

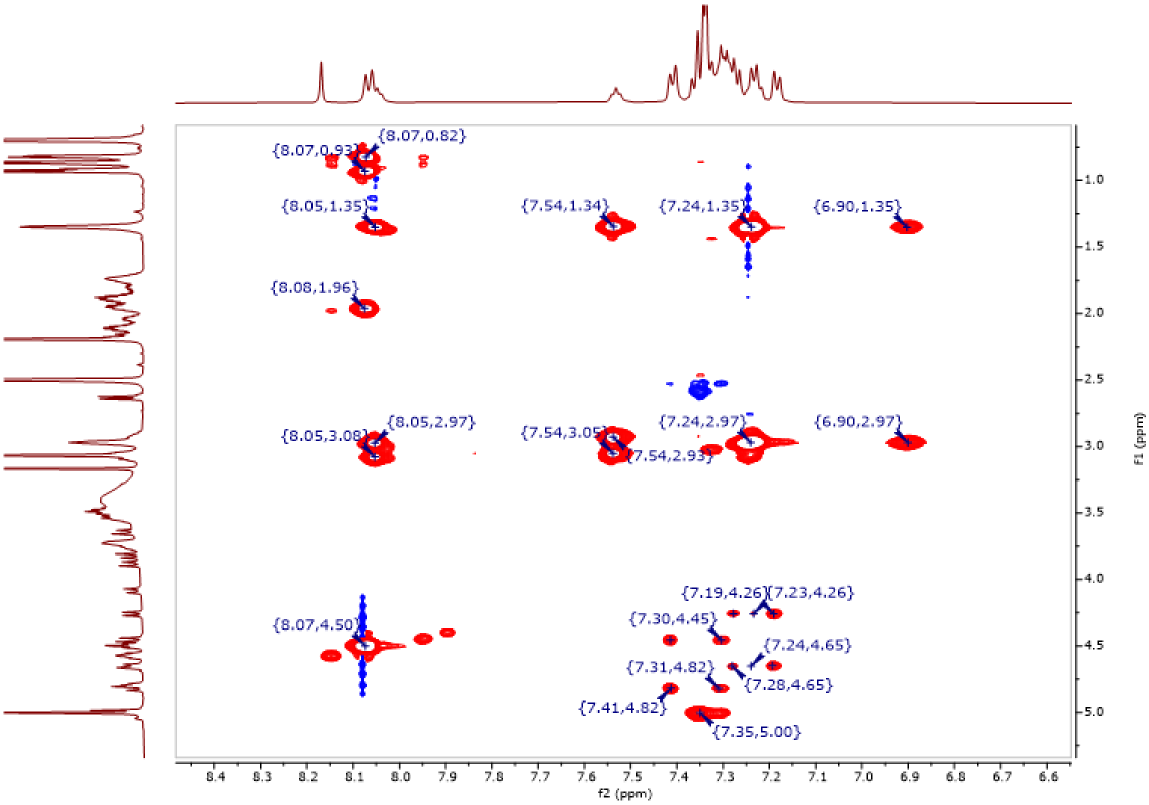

Figure S13. Fragment of the TOCSY NMR experiment of compound **5**. The signals from the *s-cis* and *s-trans* rotamers are highlighted.

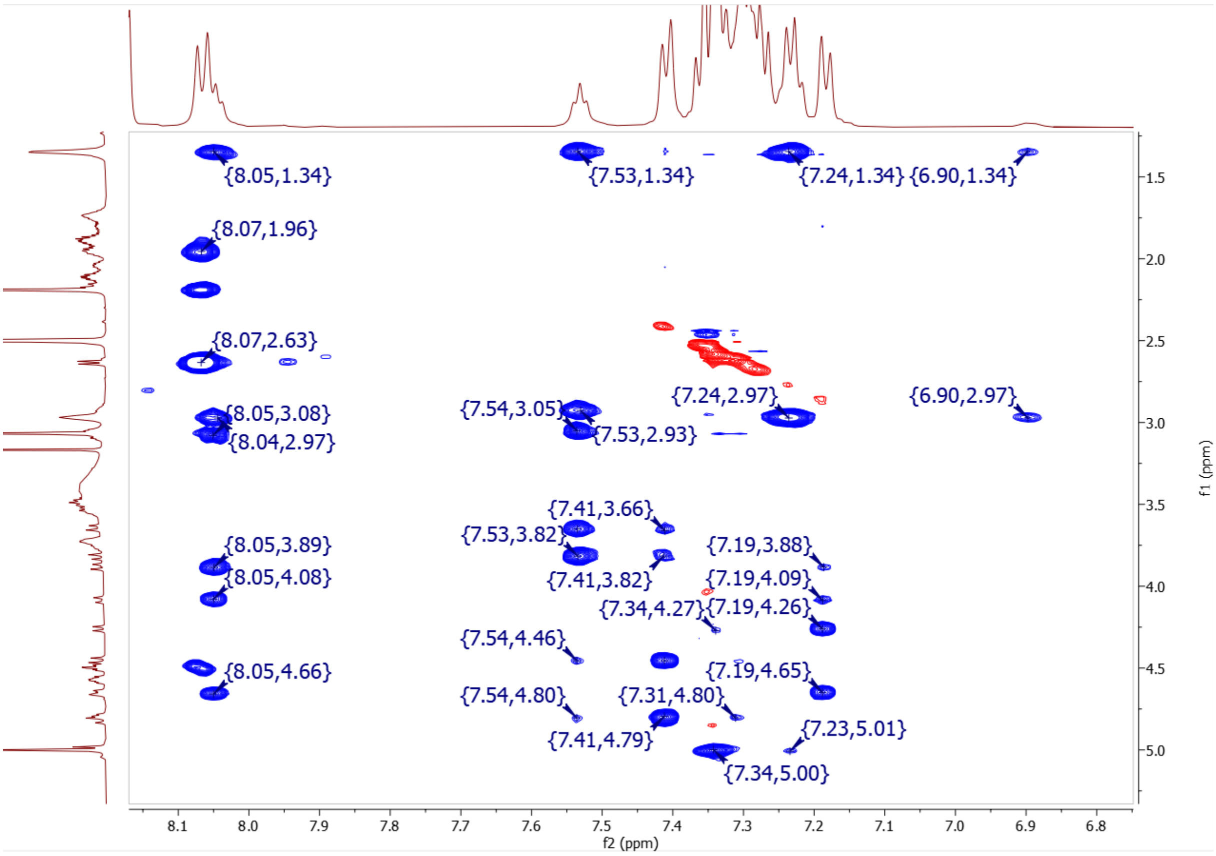

Figure S14. Fragment of the ROESY NMR experiment of compound **5**. The signals that confirm the assignment of the *s-cis* and *s-trans* rotamers are highlighted.

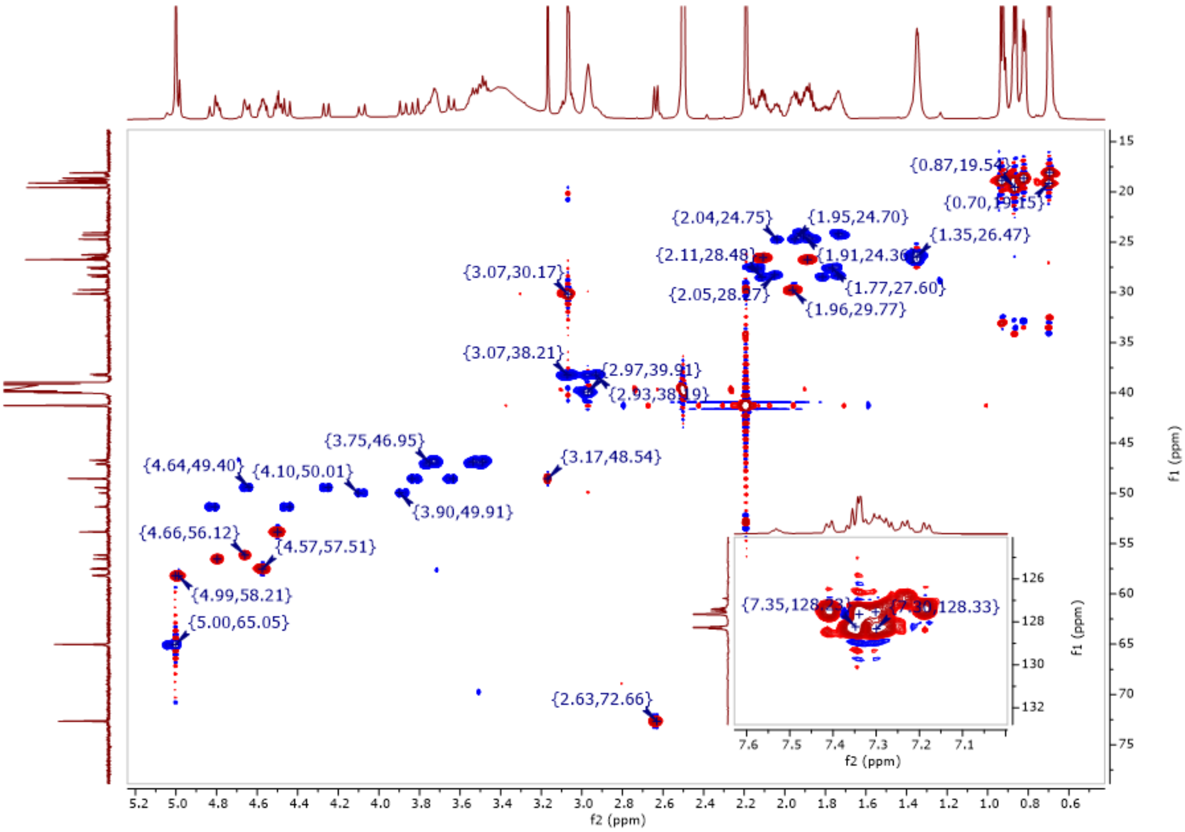

Figure S15. HSQC NMR experiment of compound **5**.

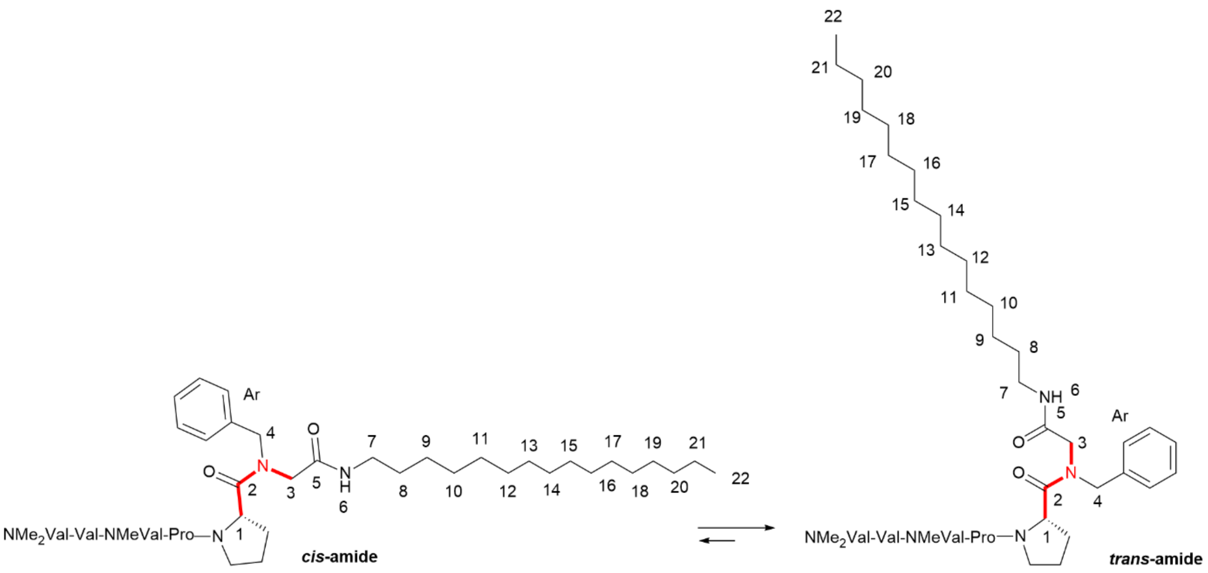

Table S6. Assignment of the ^{1}H and ^{13}C resonances of the *s-cis* and *s-trans* rotamers of compound **6**.

AA	δ_H- δ_c NH o NCH_3	δ_H- δ_c Hα-Cα	δ_H- δ_c Hβ-Cβ	δ_H- δ_c Hγ-Cγ	δ_H- δ_c Hδ-Cδ	δ_c C=O
NMe_2Val	2.20-41.3	2.63-72.7	1.89-26.8	0.88-19.6 0.71-19.2	-	170.1
Val	8.06	4.50-53.9	1.96-29.8	0.94-18.8 0.83-18.7	-	172.7
NMeVal cis	3.06-30.1	5.00-58.3	2.13-26.5	0.70-18.1 0.94-18.9	-	167.7
NMeVal trans		4.98-58.3			-	
Pro 4	-	4.60-57.5	2.17 1.76- 27.5	1.90 1.76-24.2	3.50 3.74-46.9	169.6
Pro 5 trans	-	4.81-56.5	2.03 1.73- 28.3	1.97 1.86-24.8	3.75 3.49-46.9	172.1
Pro 5 cis	-	4.67-56.2	2.14 1.81- 28.5	2.06 1.90-24.8	3.54 3.75 -46.9	172.3

Rotamer	δ_H- δ_c *s-cis*	δ_H- δ_c *s-trans*
1	4.67-56.2	4.81-56.5
2	172.3	172.1
3	3.93 4.05-50.1	3.76-48.9
4	4.62 4.30-49.4	4.82 4.50-51.5
5	167.6	167.2
6	8.02	7.44
7	3.05 2.96-38.6	3.07 2.87-38.6

Rotamer	δ_H- δ_c *s-cis*	δ_H- δ_c *s-trans*
8-21	1.34-28.9 1.26-22.1, 1.20-26.3, 1.24-29.0, 1.24-31.3	1.34-28.9 1.26-22.1, 1.20-26.3, 1.24-29.0, 1.24-31.3
22	0.88-13.9	0.93-13.9
Ar	7.19-127.4 7.24 126.9 137.2	7.31-128.3, 7.41-127.5 137.0

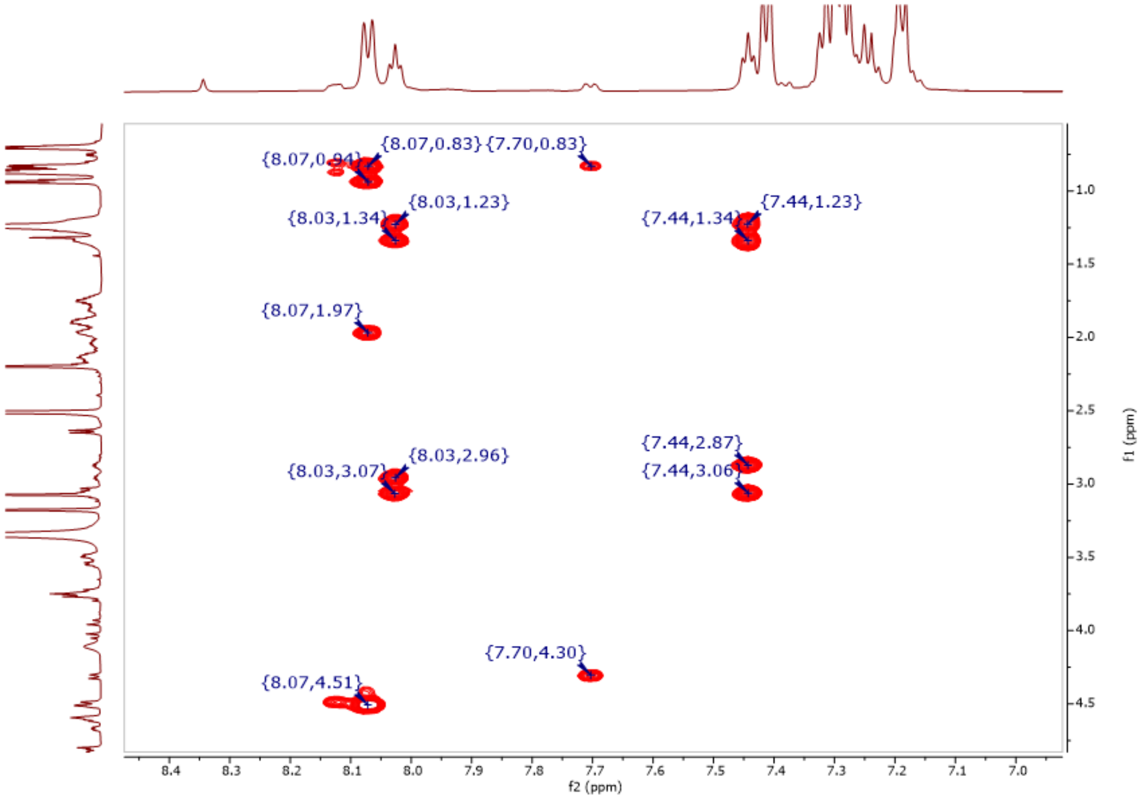

Figure S16. Fragment of the TOCSY NMR experiment of compound **6**. The signals from the *s-cis* and *s-trans* rotamers are highlighted

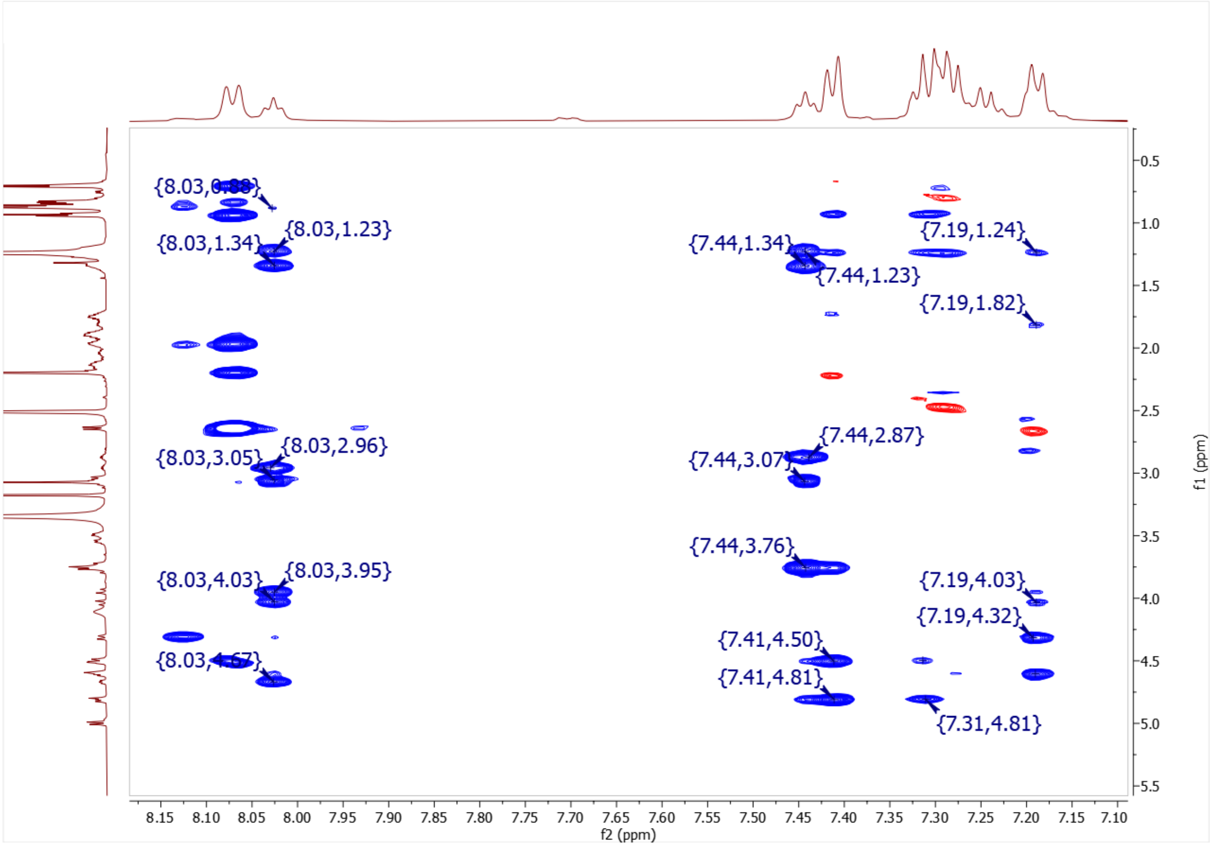

Figure S17. Fragment of the ROESY NMR experiment of compound **6**. The signals that confirm the assignment of the *s-cis* and *s-trans* rotamers are highlighted.

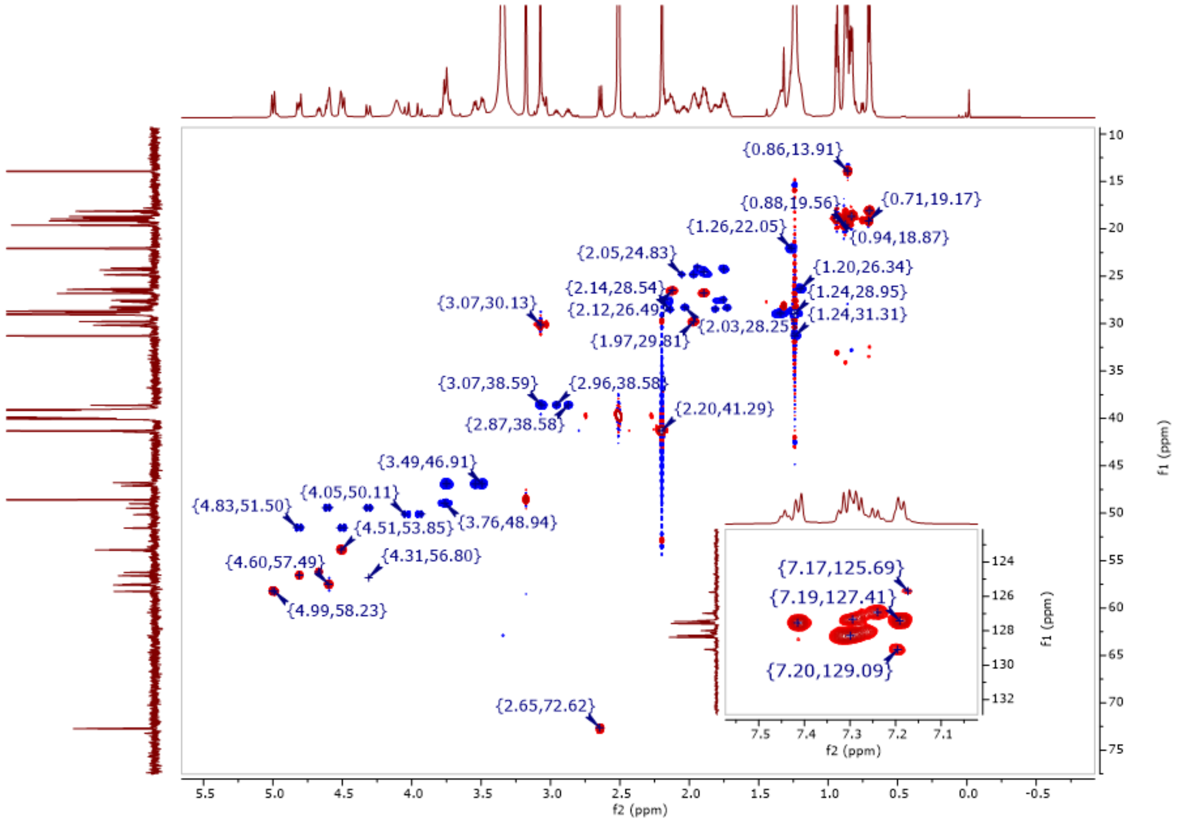

Figure S18. HSQC NMR experiment of compound **6**.

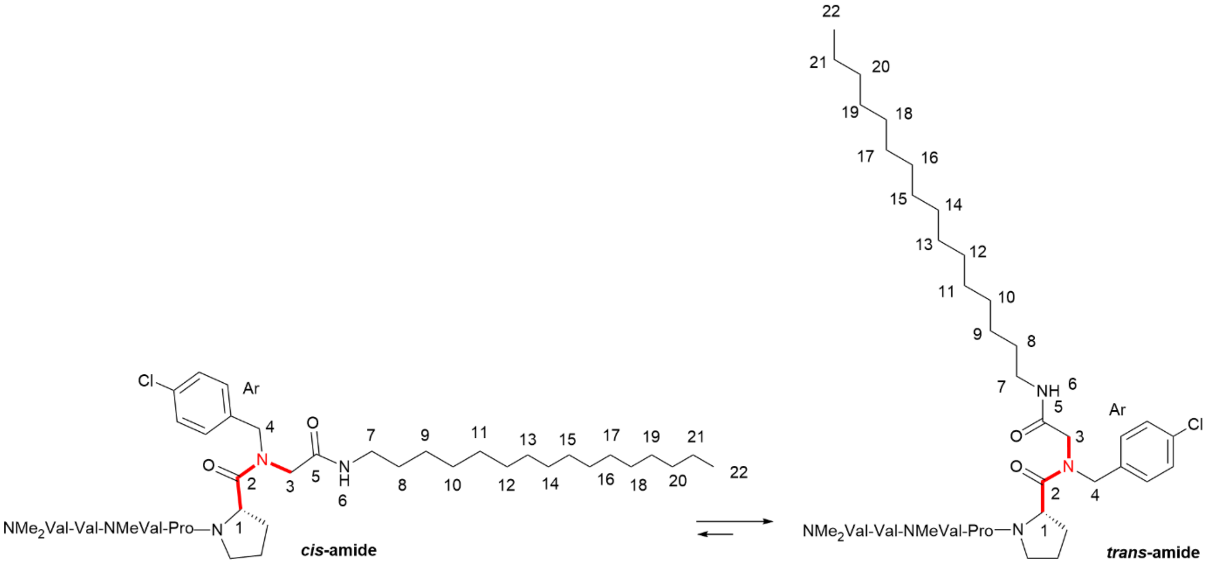

Table S7. Assignment of the ^{1}H and ^{13}C resonances of the *s-cis* and *s-trans* rotamers of compound **7**.

AA	δ_H- δ_c NH o NCH_3	δ_H- δ_c Hα-Cα	δ_H- δ_c Hβ-Cβ	δ_H- δ_c Hγ-Cγ	δ_H- δ_c Hδ-Cδ	δ_c C=O
NMe_2Val	2.20-41.8	2.65-73.2	1.90-27.2	0.87-19.9 0.72-19.1	-	170.8
Val	8.07	4.50-54.4	1.96-30.4	0.95 0.82-19.4	-	173.1
NMeVal cis	3.07-30.7	5.00-58.8	2.12-27.0	0.69 0.92-19.4	-	170.6
NMeVal trans		4.99-58.8			-	
Pro 4	-	4.59-57.9	2.17 1.76-28.0	1.91 1.75-24.7	3.74 3.49-47.5	172.0
Pro 5 trans	-	4.80-57.1	2.06 1.73-28.9	1.89 1.99-25.0	3.74 3.49-47.5	172.7
Pro 5 cis	-	4.64-56.8	2.14 2.04-28.7	1.89 1.81-25.0	3.74 3.54-47.5	172.9

Rotamer	δ_H- δ_c *s-cis*	δ_H- δ_c *s-trans*
1	4.64.56.8	4.80-57.1
2	172.9	172.7
3	4.07 3.94 -50.9	3.76-49.6
4	4.83 4.47-51.4	4.65 4.25-49.6
5	167.9	167.7
6	8.00	7.41
7	3.05 2.93-40.6	3.05 2.85-40.2

Rotamer	δ_H- δ_c *s-cis*	δ_H- δ_c *s-trans*
8-21	1.30-20.4<br>1.20-22.6<br>1.20-27.0<br>1.20-29.5<br>1.20-31.8<br>1.18-26.9	1.30-20.4<br>1.20-22.6<br>1.20-27.0<br>1.20-29.5<br>1.20-31.8<br>1.18-26.9
22	0.86-14.4	0.86-14.4
Ar	7.23-129.9<br>7.32-128.8<br>132.1,<br>136.8	7.41-128.9<br>7.46-130.1<br>132.4,<br>136.8

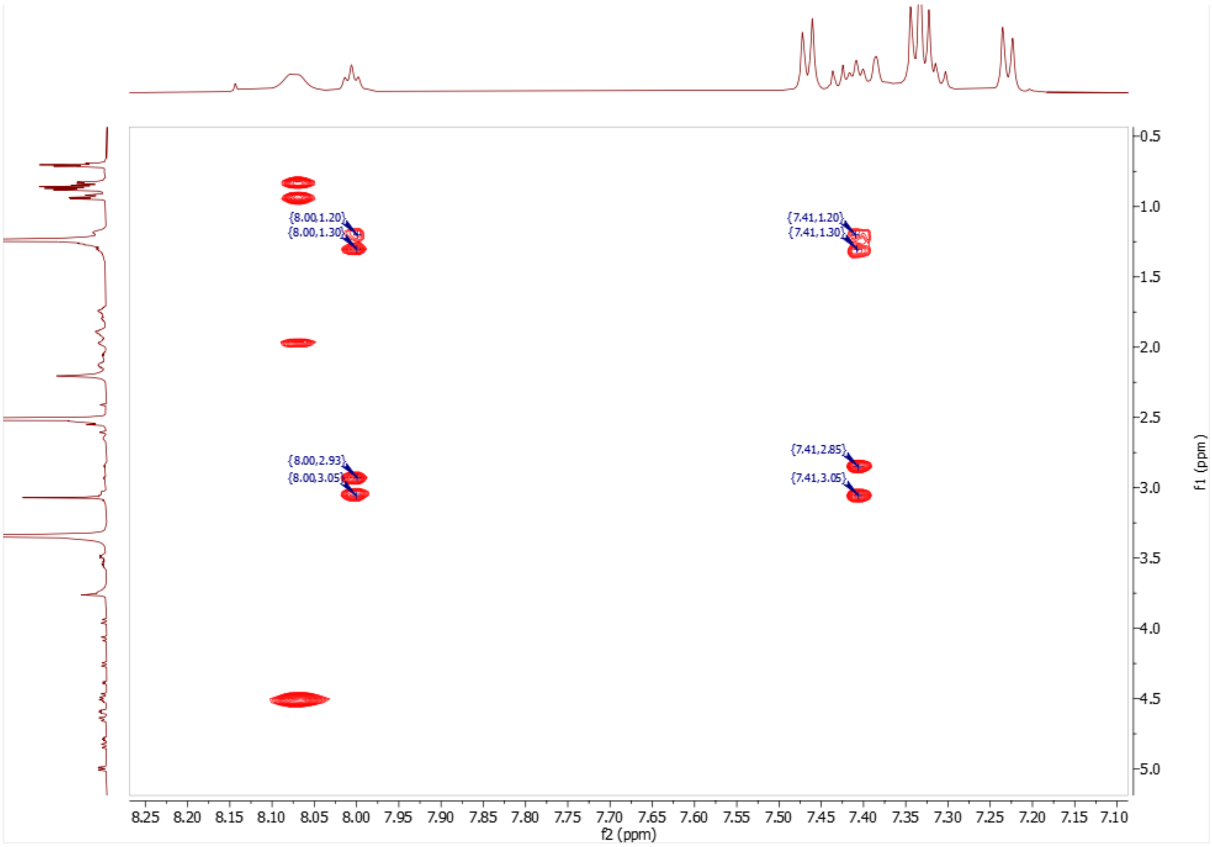

Figure S19. Fragment of the TOCSY NMR experiment of compound **7**. The signals from the *s-cis* and *s-trans* rotamers are highlighted.

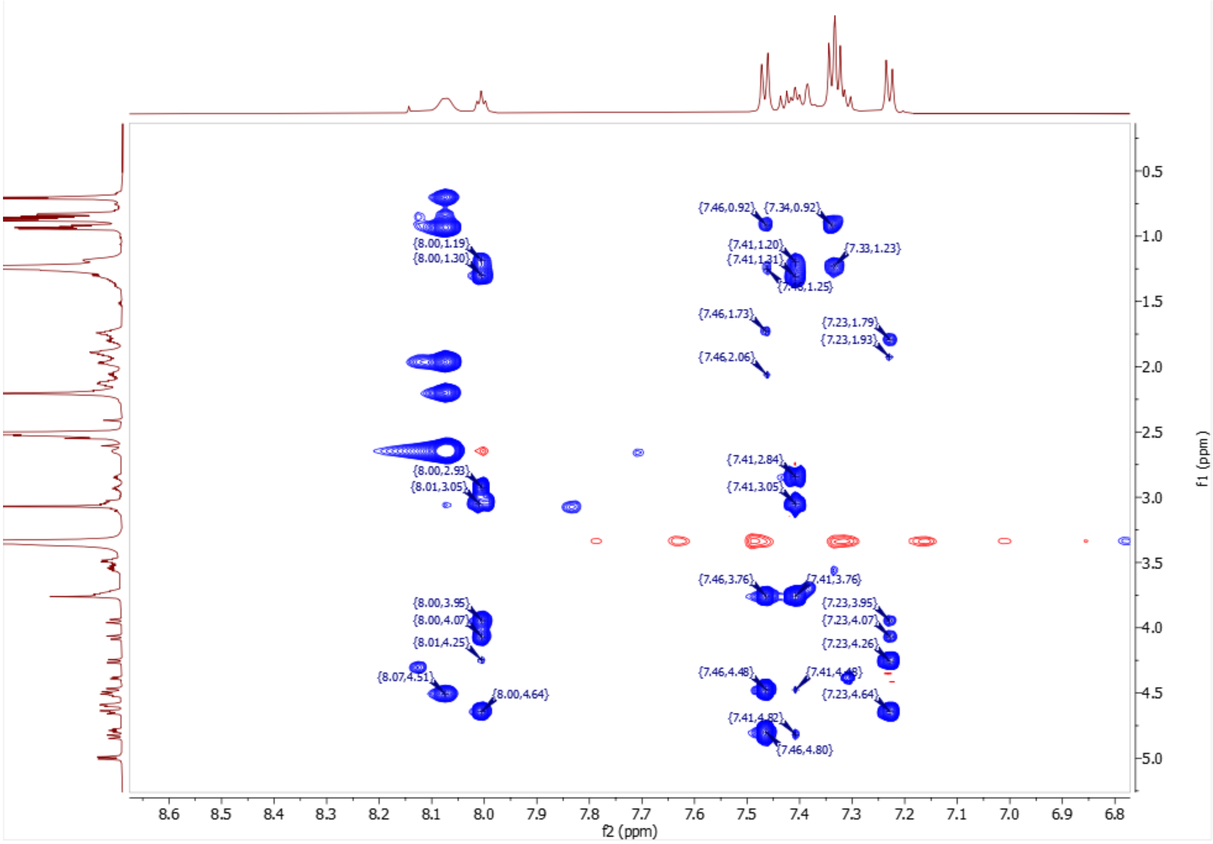

Figure S20. Fragment of the ROESY NMR experiment of compound **7**. The signals from the *s-cis* and *s-trans* rotamers are highlighted.

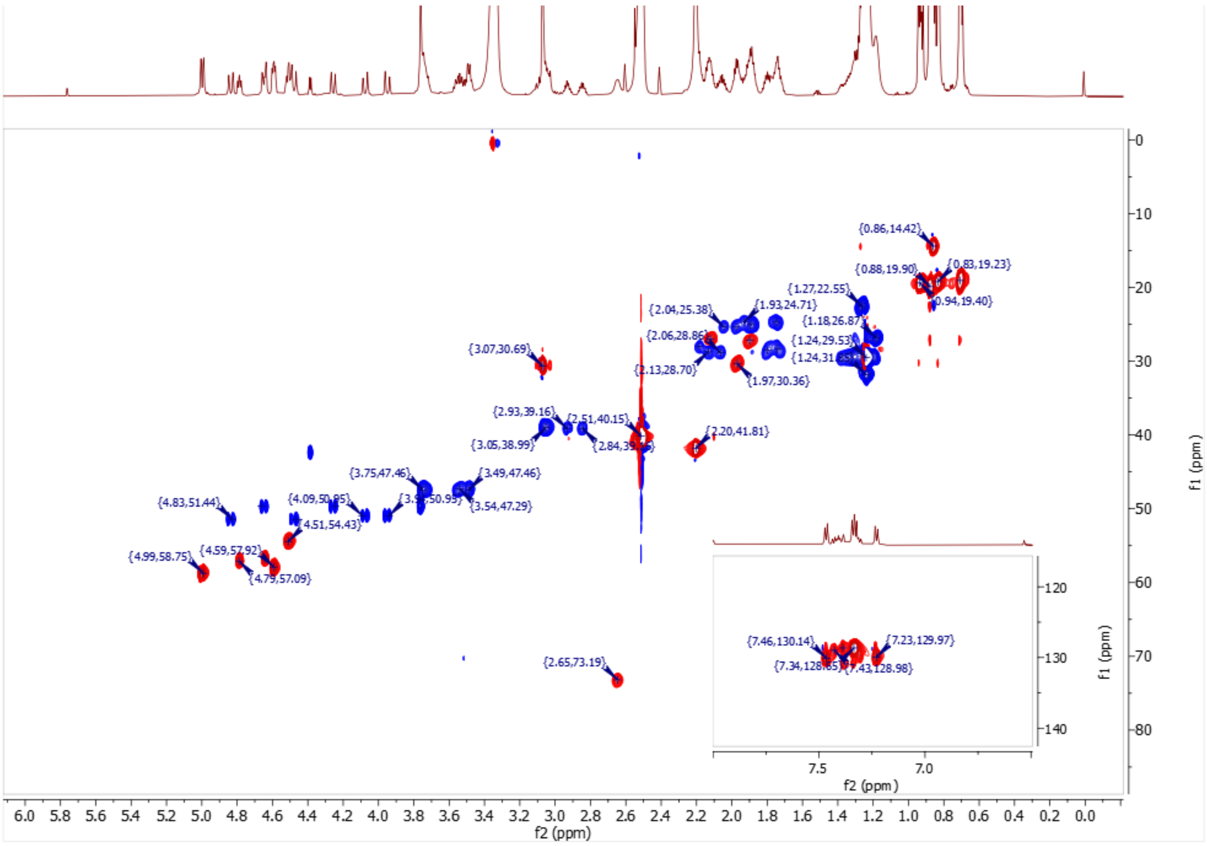

Figure S21. HSQC NMR experiment of compound **7**.

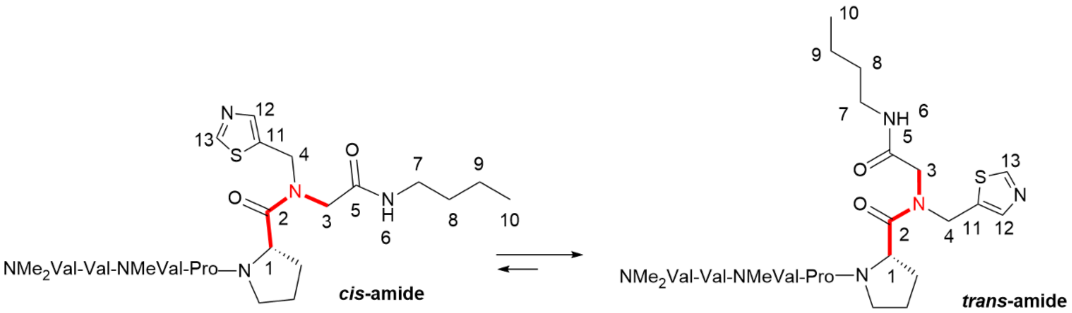

Table S8. Assignment of the ^{1}H and ^{13}C resonances of the *s-cis* and *s-trans* rotamers of compound **8**.

AA	δ_H- δ_c NH o NCH_3	δ_H- δ_c Hα-Cα	δ_H- δ_c Hβ-Cβ	δ_H- δ_c Hγ-Cγ	δ_H- δ_c Hδ-Cδ	δ_c C=O
NMe_2Val	2.50-42.4	3.00-75.1	2.14-28.7	1.00-20.0 0.87-18.5	-	171.1
Val	-	4.67-56.2	2.06-31.7	1.00 0.92-19.3	-	174.8
NMeVal cis	3.17-31.3	5.10-60.8	2.27-28.5	1.00 0.82-19.1	-	170.4
NMeVal trans	3.20-31.3	5.08-60.8	2.22-28.5	0.92 0.79-19.5	-	170.6
Pro 4	-	4.64-59.6	2.24 2.01-29.4	2.09 1.89-25.8	3.91 3.66-49.2	172.0
Pro 5 trans	-	4.96-58.4	2.16 1.90-29.2	2.14 2.04 -26.2	3.86 3.63 -48.7	174.8
Pro 5 cis	-	4.68-58.2	2.20 1.91-30.0	2.19 2.04-26.3	3.85 3.62-49.3	174.8

Rotamer	δ_H- δ_c *s-cis*	δ_H- δ_c *s-trans*
1	4.68-58.2	4.96-58.4
2	174.8	174.8
3	4.24 4.16-51.8	4.20 3.86-50.7
4	5.09 4.98-46.1	4.85 4.65-44.6
5	170.1	170.1
6	-	-
7	3.18-40.3	3.07-40.3

Rotamer	δ_H- δ_c *s-cis*	δ_H- δ_c *s-trans*
8	1.40-20.9	1.40-20.9
9	1.28-21.1	1.28-21.1
10	0.92-14.1	0.92-14.1
11	135.9	135.5
12	7.95-143.5	7.82-143.4
13	8.99-156.3	8.93-156.8

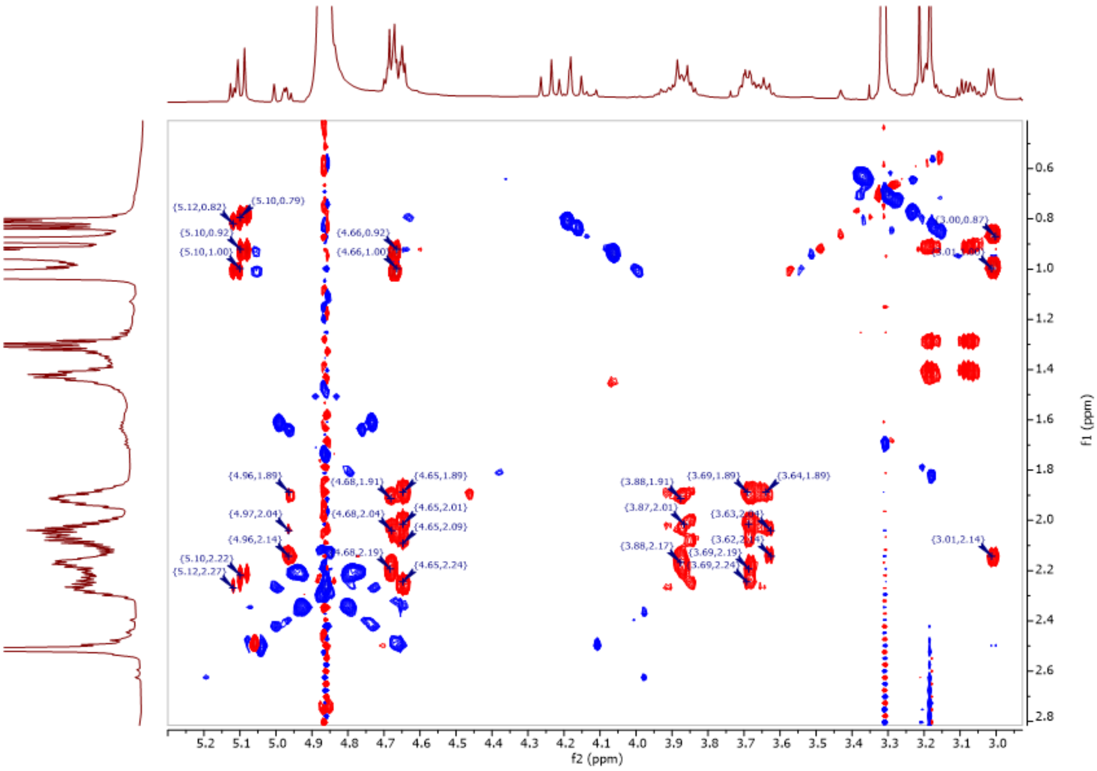

Figure S22. Fragment of the TOCSY NMR experiment of compound **8**. The signals from the *s-cis* and *s-trans* rotamers are highlighted.

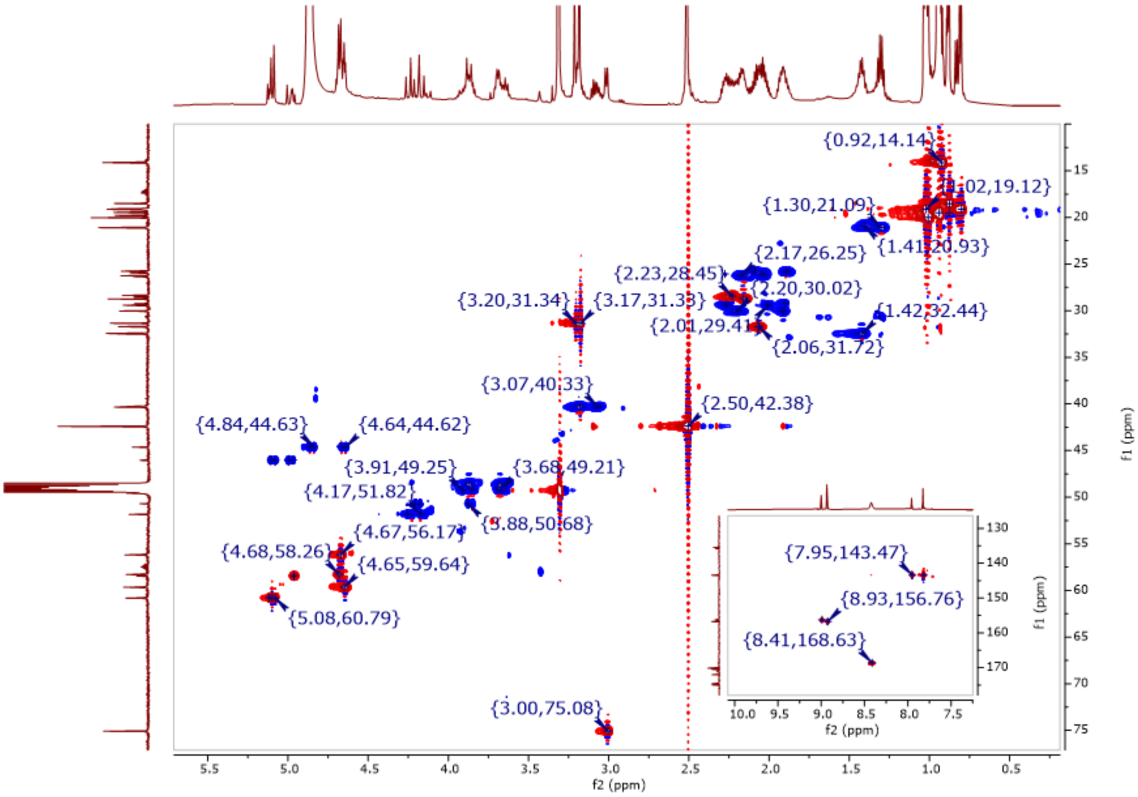

Figure S23. HSQC NMR experiment of compound **8**.

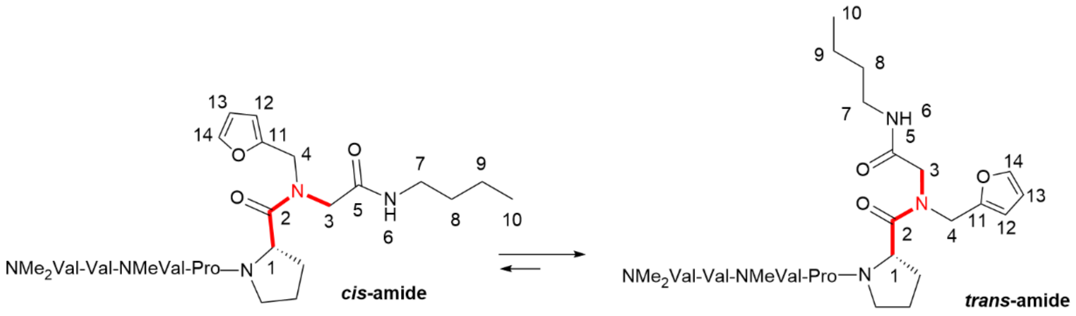

Table S9. Assignment of the ^{1}H and ^{13}C resonances of the *s-cis* and *s-trans* rotamers of compound **9**.

AA	δ_H- δ_c NH o NCH$_3$	δ_H- δ_c Hα-Cα	δ_H- δ_c Hβ-Cβ	δ_H- δ_c Hγ-Cγ	δ_H- δ_c Hδ-Cδ	δ_c C=O
NMe$_2$Val	2.76-41.6	3.72-72.1	2.28-26.5	0.84- 16.4 0.94-18.0	-	169.2
Val	8.93	4.57-54.6	2.02-30.0	0.94-18.9 0.85-18.6	-	171.6
NMeVal cis	3.08-30.2	4.99-58.2	2.16-26.5	0.89-18.7 0.71-18.4	-	167.4
NMeVal trans		4.97-58.2			-	
Pro 4	-	4.57-57.6	2.16 1.74- 27.4	1.88 1.74-24.1	3.49 3.72-46.9	169.7
Pro 5 trans	-	4.90-56.4	2.15 1.75-28.4	1.97 1.90-24.7	3.49 3.72-46.9	172.1
Pro 5 cis	-	4.61-56.3	2.09 1.74-27.2	1.98 1.89 -24.5	3.49 3.72-46.9	171.7

Rotamer	δ_H- δ_c *s-cis*	δ_H- δ_c *s-trans*
1	4.61-56.3	4.90-56.4
2	171.7	172.1
3	4.01-49.6	3.94 3.66-48.8
4	4.50 4.39-42.5	4.69 4.59-44.8
5	167.4	167.1
6	7.97	7.39
7	3.10 2.98-38.3	3.07 2.91-38.3

Rotamer	δ_H- δ_c *s-cis*	δ_H- δ_c *s-trans*
8<br>9	1.36-29.2<br>1.35-19.2<br>1.25-31.2	1.34-31.0<br>1.25-28.8<br>1.25-19.5
10	0.87-13.6	0.87-13.6
11	150.3	150.2
12	6.25-108.2	6.50-108.7
13	6.38-110.4	6.40-110.4
14	7.56-142.4	7.60-142.7

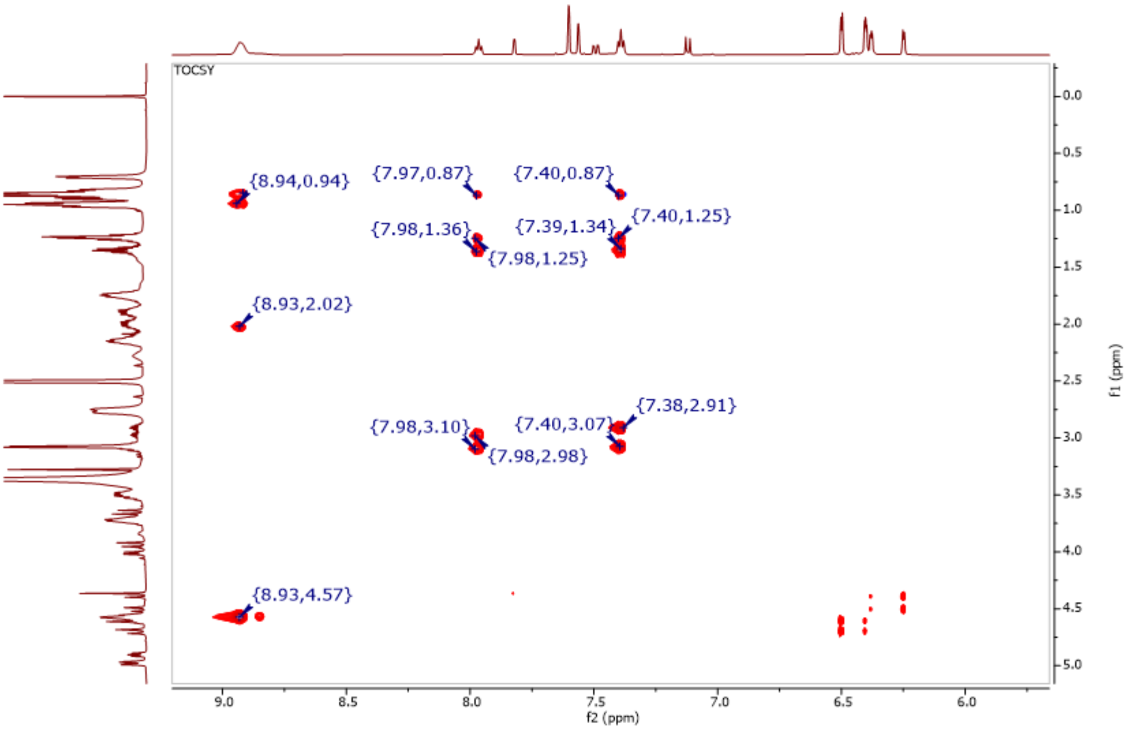

Figure S24. Fragment of the TOCSY NMR experiment of compound **9**. The signals from the *s-cis* and *s-trans* rotamers are highlighted.

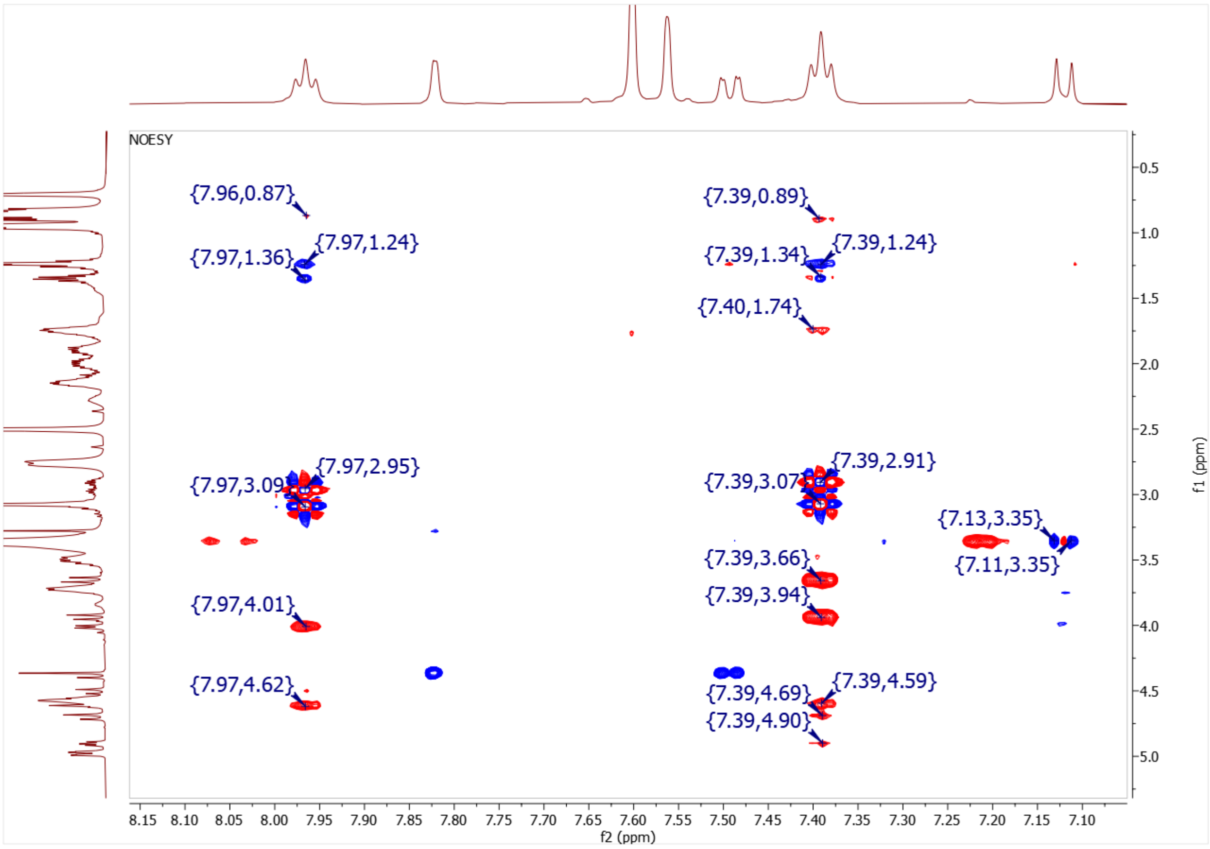

Figure S25. Fragment of the ROESY NMR experiment of compound **9**. The signals that confirm the assignment of the *s-cis* and *s-trans* rotamers are highlighted.

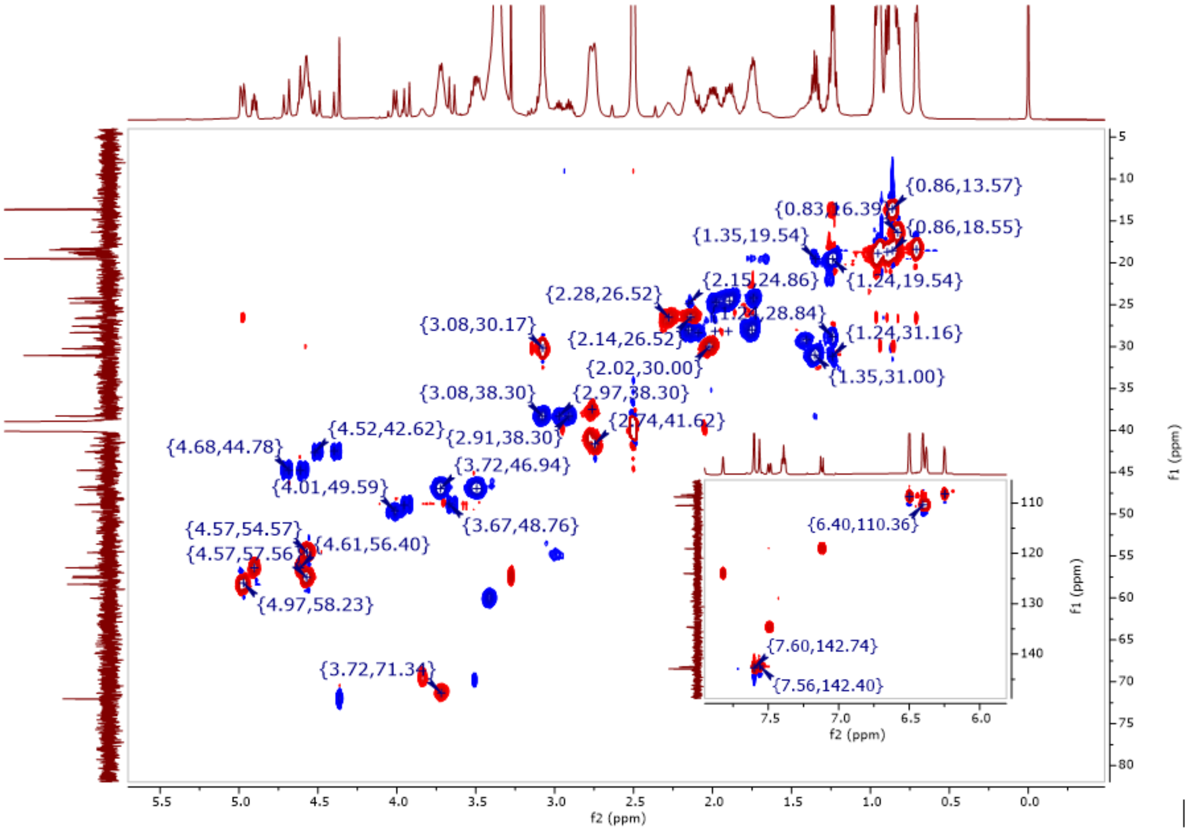

Figure S26. HSQC NMR experiment of compound **9**.

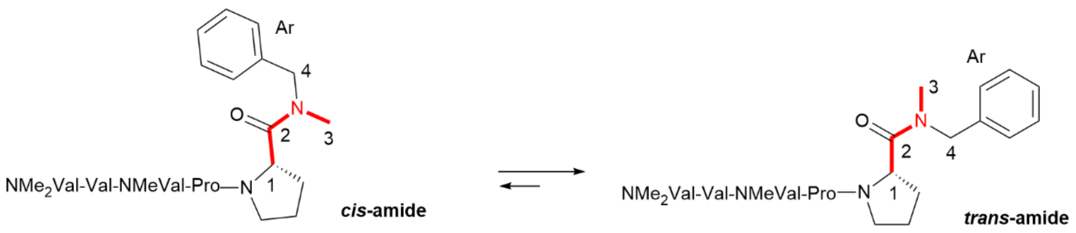

Table S10. Assignment of the ^{1}H and ^{13}C resonances of the *s-cis* and *s-trans* rotamers of compound **10**.

AA	δ_H- δ_c NH o NCH_3	δ_H- δ_c Hα-Cα	δ_H- δ_c Hβ-Cβ	δ_H- δ_c Hγ-Cγ	δ_H- δ_c Hδ-Cδ	δ_c C=O
NMe_2Val	2.50-41.8	3.02-72.5	1.93-27.2	0.89-19.7 0.70-18.5	-	172.4
Val	8.13	4.51-54.4	1.98-30.3	0.95-19.2 0.83-19.0	-	172.8
NMeVal cis	3.06-30.7	4.98-58.7	2.12-27.0	0.71-18.5 0.90-19.7	-	167.9
NMeVal trans					-	
Pro 4	-	4.57-57.9	2.17 1.72-28.1	1.93- 1.74 -24.5	3.51 3.72 -46.9	169.1
Pro 5 trans	-	4.83-56.7	2.21 1.86-28.3	1.95 1.72 -24.8	3.51 3.72 -47.1	170.6
Pro 5 cis	-	4.79-56.9	2.14 1.70-28.5	1.93 1.70-24.5	3.51 3.72 -46.9	170.6

Rotamer	δ_H- δ_c *s-cis*	δ_H- δ_c *s-trans*
1	4.79-56.9	4.83-56.7
2	170.6	170.6
3	2.70-33.5	2.95-34.8
4	4.81 4.39-52.4	4.60 4.36-50.6
Ar	137.4<br>7.42-127.4<br>7.25-127.3	137.9<br>7.31-128.8<br>7.19-127.6

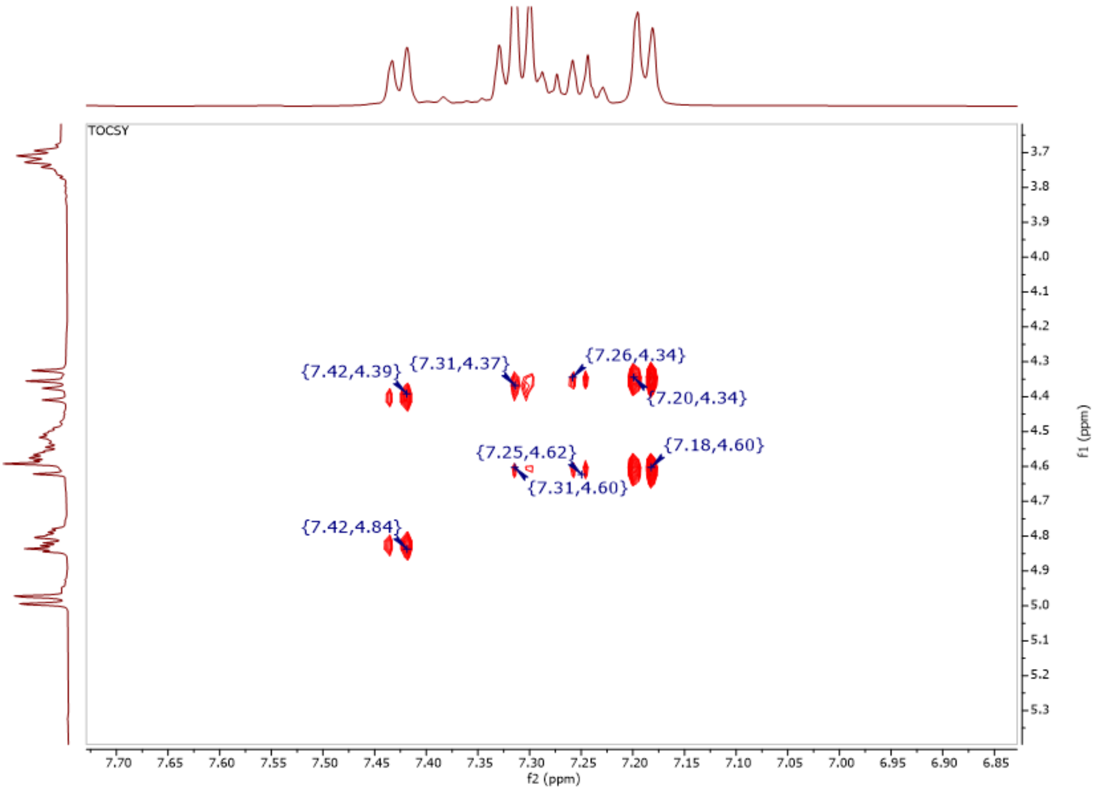

Figure S27. Fragment of the TOCSY NMR experiment of compound **10**. The signals from the *s-cis* and *s-trans* rotamers are highlighted.

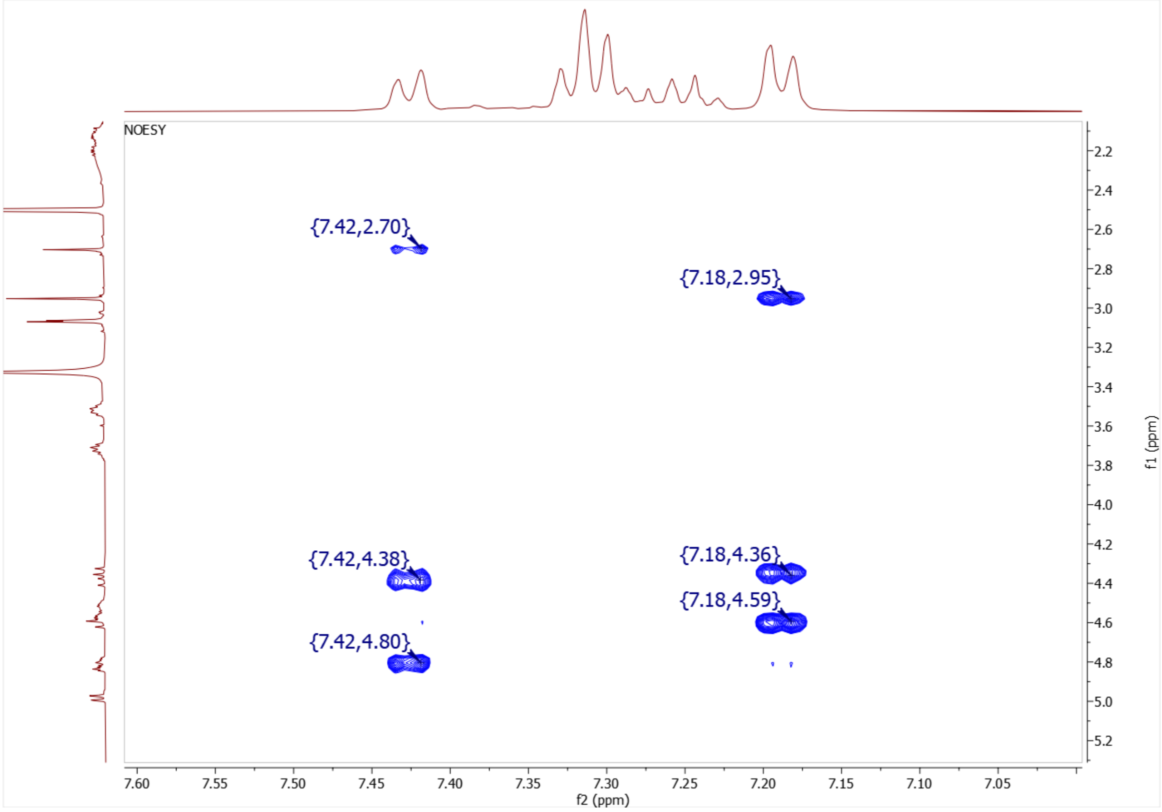

Figure S28. Fragment of the ROESY NMR experiment of compound **10**. The signals that confirm the assignment of the *s-cis* and *s-trans* rotamers are highlighted.

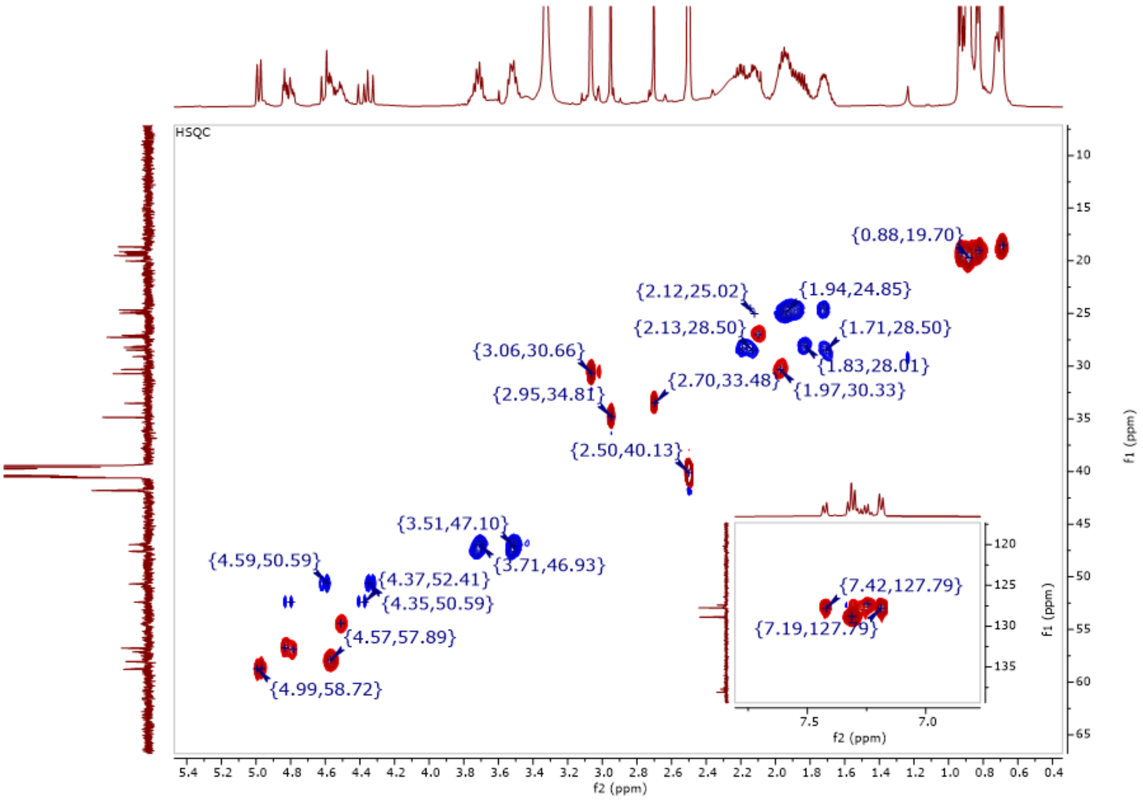

Figure S29. HSQC NMR experiment of compound **10**.

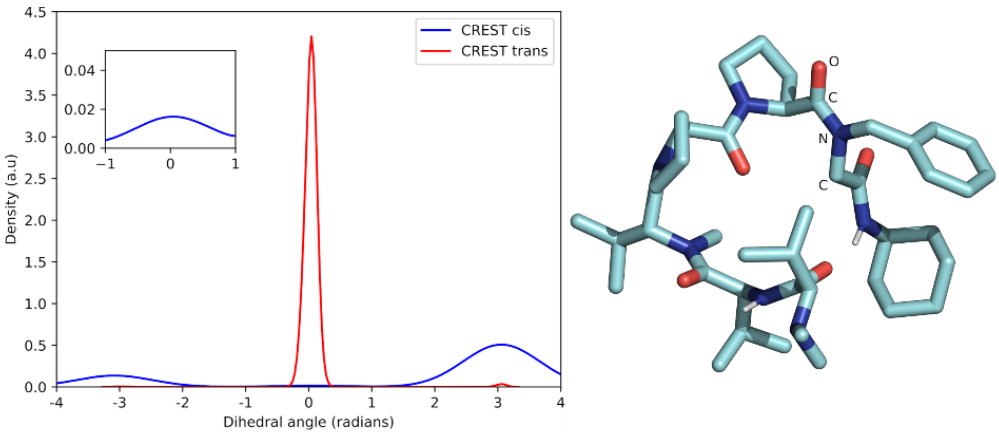

Figure S30: Conformational search with CREST. The dihedral angle between the atoms labeled on the right was used as reference to differentiate between the *s-trans* and the *s-cis* conformations. In blue and red are the distributions of dihedral angles explored using a *s-cis* and *s-trans* respectively as a starting structure.

Table S11. Binding modes, mean energy, cluster population, fingerprint-based clustering and interactions of synthetic compounds and pattern compounds with tubulin

Compound	Rotamer	Binding mode	Population	Energy (kcal/mol)	Fingerprint-based clustering	Interactions found with BINANA			
						van der Waals	Hydrogen Bond	Pi or T- staking	Salt bridge
**1**	*s-cis*	1	20	-8.38	Leaf 3	GLN(11) GLN(15) VAL(177) SER(178) ASP(179) TYR(210) PRO(222) THR(223) TYR(224) LEU(227) ALA(247) LEU(248) ASN(249) ASN(329) ILE(332) PHE(351) LYS(352) VAL(353)			
	s-trans	1	20	-8.89	Leaf 3	GLN(11) GLN(15) LYS(176) VAL(177) SER(178) ASP(179) PRO(222) THR(223) TYR(224) LEU(227) ALA(247) ASN(249) ASP(251) PRO(325) ASN(329) ILE(332) PHE(351) LYS(352) VAL(353)	ASP(179)		
**2**	*s-cis*	1	18	-8.41	Leaf 3	GLN(11) GLN(15) LYS(176) VAL(177) ASP(179) TYR(210) PRO(222) THR(223) TYR(224) LEU(227) ALA(247) LEU(248) ASN(249) ASN(329) ILE(332) PHE(351) LYS(352) VAL(353)			
	s-trans	1	20	-8.53	Leaf 3	GLN(11) GLN(15) LYS(176) VAL(177) ASP(179) TYR(210) PRO(222) THR(223) TYR(224) LEU(227) ALA(247) LEU(248) ASN(249) ASN(329) ILE(332) PHE(351) LYS(352) VAL(353)			
**3**	*s-cis*	1	9	-9.21	Leaf 9	GLN(11) LYS(176) VAL(177) SER(178) ASP(179) TYR(210) PRO(222) THR(223) TYR(224) LEU(248) PRO(325) ASN(329) ILE(332) LYS(352) VAL(353) ILE(355)		TYR(224)	GLU(254)
		2	4	-9.05	Leaf 5	GLN(11) LYS(176) VAL(177) ASP(179) TYR(210) PRO(222) THR(223) TYR(224) LEU(227) ALA(247) LEU(248) ASN(249) PRO(325) ASN(329) ILE(332) PHE(351) LYS(352) VAL(353) ILE(355)			

		3	4	-9.30	Leaf 12	PRO(175) LYS(176) VAL(177) ASP(179) TYR(210) PRO(222) THR(223) TYR(224) LEU(248) PRO(325) ASN(329) ILE(332) PHE(351) VAL(353)			
	s-trans	1	20	-9.21	Leaf 3	GLN(11) PRO(175) LYS(176) VAL(177) SER(178) ASP(179) TYR(210) PRO(222) THR(223) TYR(224) LEU(227) ALA(247) LEU(248) ASN(329) ILE(332) PHE(351) LYS(352) VAL(353)			
**4**	*s-cis*	1	20	-10.21	Leaf 12	PRO(175) LYS(176) VAL(177) ASP(179) TYR(210) PRO(222) THR(223) TYR(224) LEU(227) LEU(248) PRO(325) ASN(329) ILE(332) PHE(351) VAL(353)	PRO(222)		
	s-trans	1	17	-8.78	Leaf 4	GLN(15) LYS(176) VAL(177) TYR(210) PRO(222) THR(223) TYR(224) LEU(227) ALA(247) LEU(248) ASN(249) PRO(325) LYS(352) VAL(353)			
		2	3	-8.70	Leaf 9	PRO(175) LYS(176) VAL(177) ASP(179) PRO(222) THR(223) TYR(224) ALA(247) LEU(248) ASN(249) PRO(325) ASN(329) ILE(332) PHE(351) LYS(352) VAL(353)			GLU(254)
**5**	*s-cis*	1	9	-9.01	Leaf 8	GLN(11) LYS(176) VAL(177) ASP(179) TYR(210) PRO(222) THR(223) TYR(224) LEU(227) ALA(247) LEU(248) ASN(249) PRO(325) ASN(329) ILE(332) PHE(351) VAL(353)	ALA(247)		
		2	4	-9.45	Leaf 0	GLN(11) PRO(175) LYS(176) VAL(177) SER(178) ASP(179) TYR(210) PRO(222) TYR(224) LEU(248) PRO(325) ASN(329) ILE(332) LYS(352) VAL(353) ILE(355)			GLU(254)
		3	3	-8.90	Leaf 12	GLN(11) GLN(15) LYS(176) VAL(177) SER(178) ASP(179) TYR(210) PRO(222) THR(223) TYR(224) LEU(227) ALA(247)			

						LEU(248) ASN(249) PRO(325) ASN(329) ILE(332) LYS(352) VAL(353)			
	s-trans	1	13	-9.06	Leaf 10	GLN(11) GLN(15) LYS(176) VAL(177) SER(178) ASP(179) TYR(210) PRO(222) THR(223) TYR(224) LEU(227) ALA(247) LEU(248) ASN(249) ASN(329) ILE(332) LYS(352) VAL(353)			
		2	7	-9.17	Leaf 0	GLN(11) GLN(15) LYS(176) VAL(177) SER(178) ASP(179) PRO(222) THR(223) TYR(224) ALA(247) LEU(248) ASN(249) PRO(325) ASN(329) ILE(332) PHE(351) LYS(352) VAL(353) ILE(355)	VAL(177)		
**6**	*s-cis*	1	13	-7.60	Leaf 8	GLN(11) GLN(15) VAL(177) SER(178) ASP(179) TYR(210) PRO(222) THR(223) TYR(224) LEU(227) ALA(247) LEU(248) ASN(249) PRO(325) ASN(329) ILE(332) PHE(351) LYS(352) VAL(353) ILE(355)			
		2	6	-8.17	Leaf 4	GLN(11) GLN(15) VAL(177) SER(178) ASP(179) PRO(222) THR(223) TYR(224) LEU(227) ALA(247) LEU(248) ASN(249) PRO(325) ASN(329) LYS(352) VAL(353) ILE(355)	GLN(11)		
	s-trans	1	14	-7.46	Leaf 4	GLN(11) GLN(15) VAL(177) SER(178) ASP(179) TYR(210) PRO(222) THR(223) TYR(224) LEU(227) ALA(247) LEU(248) ASN(249) PRO(325) ASN(329) LYS(352) VAL(353) ILE(355)			
		2	4	-7.40	Leaf 7	GLN(11) PRO(175) LYS(176) VAL(177) SER(178) ASP(179) PRO(222) THR(223) TYR(224) ALA(247) LEU(248) PRO(325) ASN(329) ILE(332) PHE(351) LYS(352) VAL(353) ILE(355)	ALA(247)		
**7**	*s-cis*	1	14	-7.15	Leaf 4	GLN(11) GLN(15) VAL(177) SER(178) ASP(179) PRO(222) THR(223) TYR(224) LEU(227) ALA(247) LEU(248) ASN(249)	GLN(11)		

						PRO(325) ASN(329) LYS(352) VAL(353) ILE(355)			
		2	3	-7.33	Leaf 8	GLN(11) GLN(15) PRO(175) LYS(176) VAL(177) ASP(179) TYR(210) PRO(222) THR(223) TYR(224) LEU(227) ALA(247) ASN(249) PRO(325) ASN(329) ILE(332) PHE(351) LYS(352) VAL(353)		TYR(224)	
	s-trans	1	13	-7.69	Leaf 12	GLN(11) PRO(175) LYS(176) VAL(177) SER(178) ASP(179) TYR(210) PRO(222) THR(223) TYR(224) LEU(227) ALA(247) LEU(248) ASN(249) ASN(329) ILE(332) PHE(351) LYS(352) VAL(353)	ASN(329)	TYR(224)	
		2	6	-7.75	Leaf 5	GLN(11) GLN(15) VAL(177) SER(178) ASP(179) TYR(210) PRO(222) THR(223) TYR(224) LEU(227) ALA(247) LEU(248) ASN(249) PRO(325) ASN(329) ILE(332) PHE(351) LYS(352) VAL(353) ILE(355)			
**8**	*s-cis*	1	19	-7.84	Leaf 4	GLN(11) GLN(15) VAL(177) SER(178) ASP(179) PRO(222) THR(223) TYR(224) LEU(227) LEU(248) ASN(249) PRO(325) ASN(329) LYS(352) VAL(353) ILE(355)	GLN(11)		
	s-trans	1	17	-7.61	Leaf 4	GLN(11) VAL(177) PRO(222) THR(223) TYR(224) ALA(247) LEU(248) PRO(325) ASN(329) LYS(352) VAL(353) ILE(355)			GLU(254)
		2	3	-7.73	Leaf 3	GLN(11) GLN(15) LYS(176) VAL(177) SER(178) ASP(179) PRO(222) THR(223) TYR(224) ALA(247) LEU(248) ASN(249) ASN(329) ILE(332) PHE(351) LYS(352) VAL(353)	ASP(179)		
**9**	*s-cis*	1	20	-8.06	Leaf 3	GLN(11) GLN(15) LYS(176) VAL(177) ASP(179) PRO(222) THR(223) TYR(224) LEU(227) ALA(247) LEU(248) ASN(249) ASN(329) LYS(352) VAL(353)			
	s-trans	1	11	-7.91	Leaf 1	GLN(11) GLN(15) LYS(176) VAL(177) SER(178) ASP(179) TYR(210) PRO(222)		TYR(224)	

						THR(223) TYR(224) ALA(247) LEU(248) ASN(329) PHE(351) LYS(352) VAL(353)			
		2	9	-7.95	Leaf 4	GLN(11) VAL(177) PRO(222) THR(223) TYR(224) ALA(247) LEU(248) PRO(325) ASN(329) LYS(352) VAL(353) ILE(355)			GLU(254)
**10**	*s-cis*	1	14	-8.71	Leaf 9	GLN(11) PRO(175) LYS(176) VAL(177) SER(178) ASP(179) PRO(222) TYR(224) ALA(247) LEU(248) PRO(325) ASN(329) ILE(332) PHE(351) LYS(352) ILE(355)			GLU(254)
		2	6	-8.65	Leaf 2	GLN(11) LYS(176) VAL(177) SER(178) ASP(179) PRO(222) THR(223) TYR(224) ALA(247) LEU(248) ASN(249) ASN(329) LYS(352) VAL(353)		TYR(224)	GLU(254)
	s-trans	1	20	-8.60	Leaf 2	GLN(11) GLN(15) LYS(176) VAL(177) SER(178) ASP(179) TYR(210) PRO(222) THR(223) TYR(224) LEU(227) ALA(247) LEU(248) ASN(329) LYS(352) VAL(353)		TYR(224)	
**cemadotin**	-	1	18	-8.35		LYS(176) VAL(177) ASP(179) TYR(210) TYR(224) PRO(325) ASN(329) ILE(332)	ASN(329)		
**Dolastatin 15**	-	1	14	-8.27	Leaf 6	GLN(11) LYS(176) VAL(177) SER(178) ASP(179) TYR(210) THR(223) TYR(224) LEU(227) ALA(247) ASN(249) PRO(325) ASN(329) LYS(352) VAL(353)			
		2	5	-8.34	Leaf 11	LYS(176) VAL(177) SER(178) ASP(179) TYR(210) THR(223) TYR(224) PRO(325) ASN(329) ILE(332) LYS(352) VAL(353) ILE(355)			

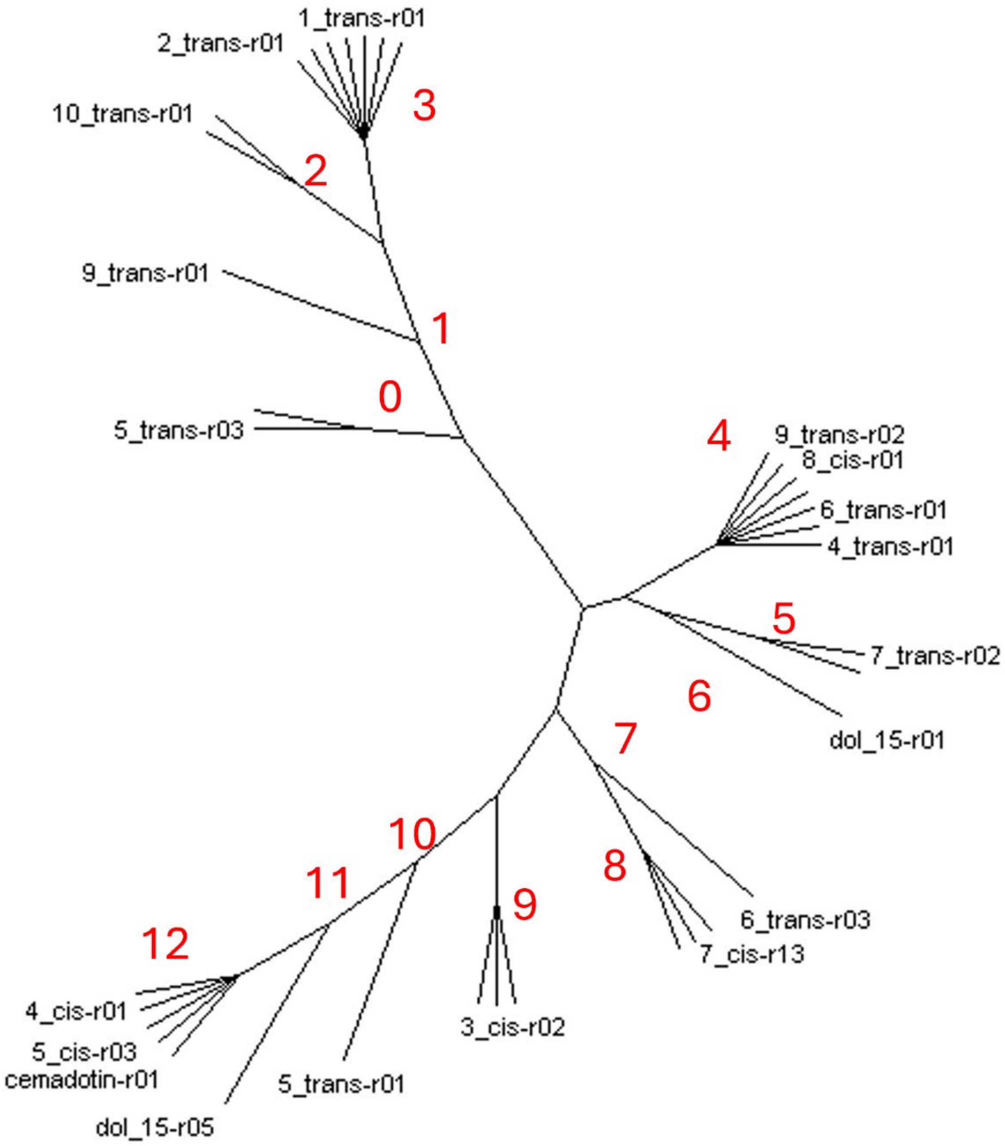

Figure S31. Hierarchical tree map where docked compounds are clustered based on their contact fingerprint towards tubulin. Compounds docked together have a similar interaction pattern. Each cluster (0-12) is highlighted by a red number at each node.

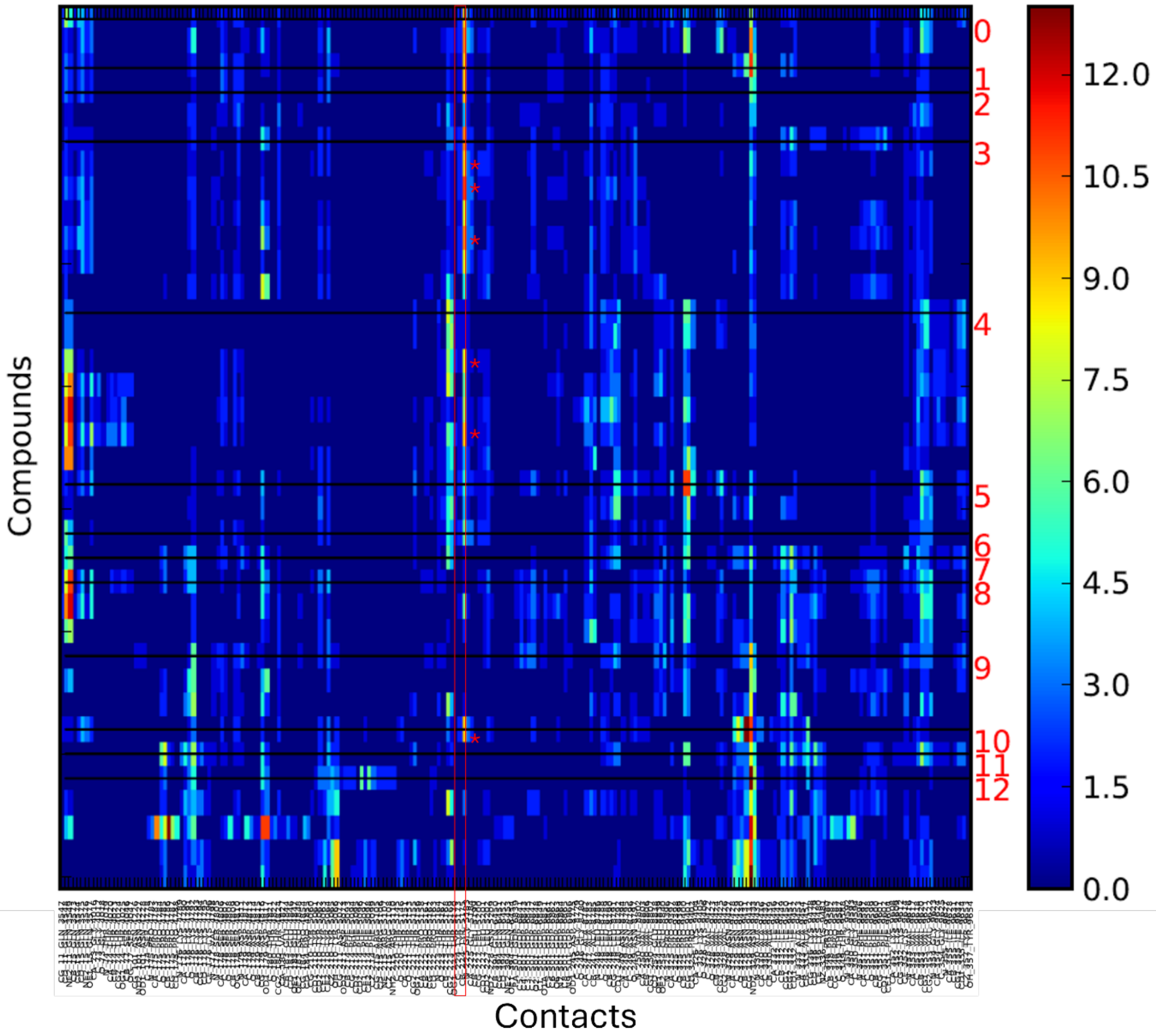

Figure S32. Contact map of the docked compounds (s-cis and s-trans conformations) which are clustered based on their contact fingerprint (Y axis) towards tubulin sequence (X axis). The color scale indicates the contact intensity average where the dark blue color corresponds to a low-intensity contact (close to zero) and in this method groups are separated by black lines. Contacts with βTyr224 is highlighted as red rimmed box. * Interaction intensity with β:Tyr224 reached 8-11 units.

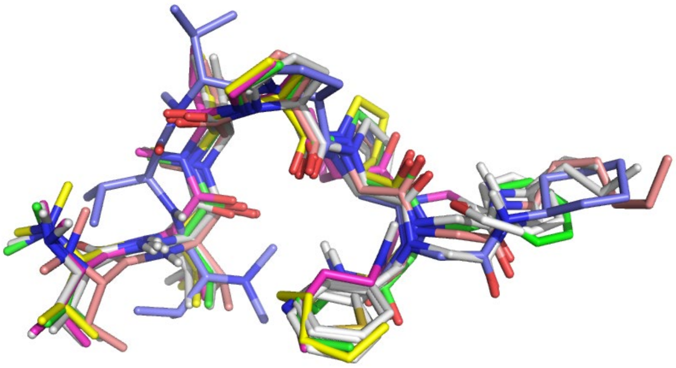

Figure S33. Overlay of 3D structures of the docked compounds of group 3 (Compounds **1**_*s-trans*, **1**_*s-cis*, **9**_*s-cis*, **2**_*s-cis*, **2**_*s-trans*, **8**_*s-trans*, **3**_*s-trans*), for which a conserved binding mode is observed, independently of the configuration being *s-cis* or *s-trans*.

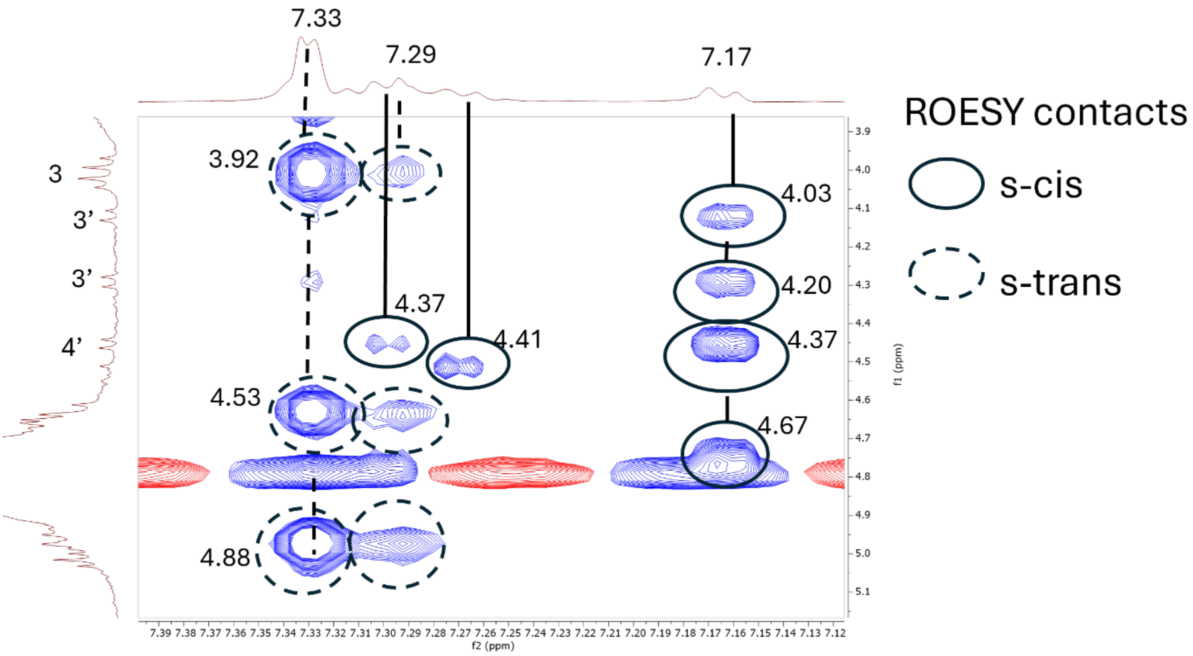

Figure S34. ROESY NMR spectra of compound **1** in 10 mM sodium phosphate buffer pD 7.2 in 99.9% D_2O. *S-cis* and *s-trans* systems are observed.

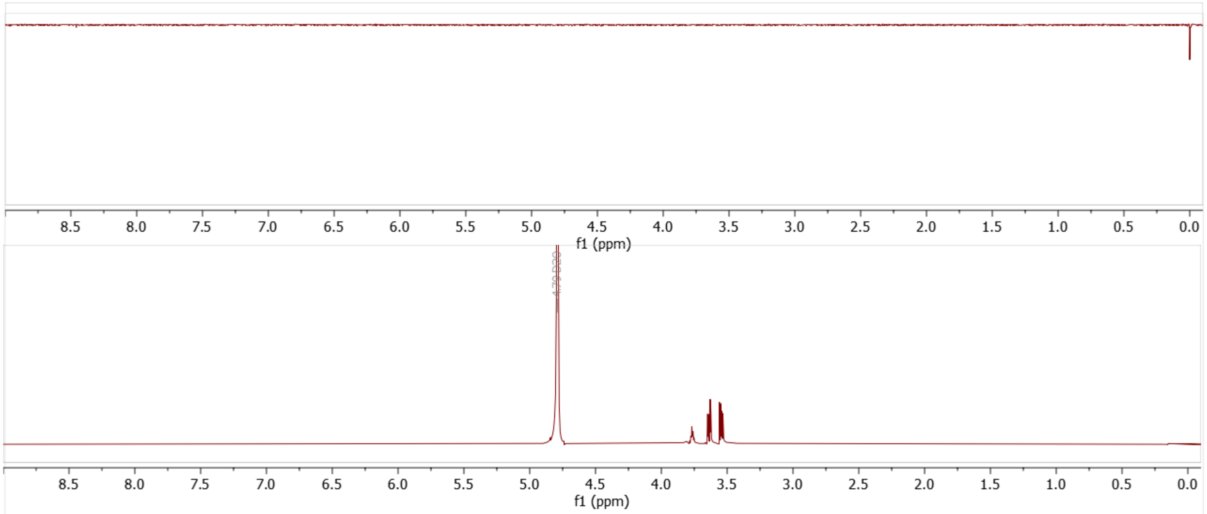

Figure S35. Control experiment for the ligand, compound **1** (upper spectrum) where no signal is detected as expected and tubulin (lower spectrum) where only the signals from the co-factor are detected at the tested concentration.

Table S12. Epitope mapping calculations for the mixture of conformers of compound **1**.

	Range (ppm)	Absolute intensity of the off-resonance spectrum	Absolute intensity of the difference spectrum	STD intensity	Epitope mapping
1	7.36 - 7.32	1.31813E+13	3.76358E+12	0.286	100%
2	7.32 - 7.25	1.21755E+13	3.26736E+12	0.268	94%
3	7.19 - 7.14	4.16857E+12	9.46053E+11	0.227	79%
4	3.98 - 3.85	1.59171E+13	1.33411E+12	0.084	29%
5	3.84 - 3.70	3.19653E+13	1.65145E+12	0.052	18%
6	3.69 - 3.60	1.79719E+13	9.38822E+11	0.052	18%
7	3.60 - 3.52	2.19155E+13	8.06353E+11	0.037	13%
8	3.16 - 3.08	2.35058E+13	2.8732E+12	0.122	43%

9	3.07 - 2.94	1.36619E+13	1.8891E+12	0.138	48%
10	2.52 - 2.41	3.58104E+13	2.51462E+12	0.070	25%
11	2.32 -2.19	9.46163E+12	1.13861E+12	0.120	42%
12	2.19 - 2.07	1.62696E+13	2.35165E+12	0.145	51%
13	2.02 - 1.91	2.30119E+13	3.55394E+12	0.154	54%
14	1.91 - 1.75	1.99616E+13	2.42365E+12	0.121	43%
15	1.34 - 1.24	1.37535E+13	2.33213E+12	0.170	59%
16	1.19 - 1.09	1.41237E+13	2.57272E+12	0.182	64%
17	0.97 - 0.85	5.92615E+13	1.03546E+13	0.175	61%
18	0.85 - 0.79	2.37783E+13	3.73891E+12	0.157	55%
19	0.79 - 0.70	5.43137E+13	1.21913E+13	0.225	79%

*registered at 2s saturation time
